# Towards High‐Throughput Testing of Bipolar Plate Materials for Proton Exchange Membrane Water Electrolyzers: Correlative Contact Resistance and Stability Analysis

**DOI:** 10.1002/smtd.71010

**Published:** 2026-09-03

**Authors:** Lena Fiedler, Andreas Körner, George Pätzold, Elaheh Akbarnejad, Alan Savan, Simon Thiele, Dominik Dworschak, Alfred Ludwig, Karl J. J. Mayrhofer, Andreas Hutzler

**Affiliations:** ^1^ Helmholtz Institute Erlangen‐Nürnberg for Renewable Energy (IET‐2) Forschungszentrum Jülich GmbH Erlangen Germany; ^2^ Department of Chemical and Biological Engineering Friedrich‐Alexander‐Universität Erlangen‐Nürnberg Erlangen Germany; ^3^ Materials Discovery and Interfaces, Institute for Materials Ruhr‐Universität Bochum Bochum Germany

**Keywords:** corrosion, dissolution, interfacial contact resistance, platinum coatings, water electrolysis

## Abstract

Bipolar plates (BPP) are a major cost driver in proton exchange membrane water electrolyzers for green hydrogen production that require replacement with more cost‐effective materials. However, novel materials must combine high corrosion resistance and low dissolution rates with low interfacial contact resistance (ICR). Herein, we introduce a correlative workflow for determining electrochemical dissolution using a scanning flow cell coupled on‐line to an inductively coupled plasma spectrometer and ICR, utilizing a novel miniaturized ICR setup with a sample diameter of 2 mm. The applicability of the setup is demonstrated using titanium (Ti), for which the ICR is found to increase depending on the applied oxidation potential. Furthermore, automated ICR mapping across pristine Ti sheets with a platinum (Pt) coating featuring a thickness gradient reveals a pronounced ICR decrease when the Pt layer exceeds ∼3.8 ± 1.4 nm_Pt_, while thicker Pt coatings (up to ∼80 nm_Pt_) do not provide further improvement. These results demonstrate that ultrathin Pt coatings can already substantially reduce the ICR of titanium‐based BPP materials. Our setup enables high‐throughput screening of material libraries and provides a powerful tool for developing optimized PEMWE components with low ICR and high electrochemical stability.

## Graphical abstract

Bipolar plates are a major cost driver in proton exchange membrane water electrolyzers (PEMWE) and require novel cost‐effective materials combining high corrosion resistance with low interfacial contact resistance (ICR). We present a correlative workflow for spatially resolved dissolution and ICR measurements by developing a miniaturized ICR measurement setup providing a powerful high‐throughput approach for identifying novel PEMWE materials.

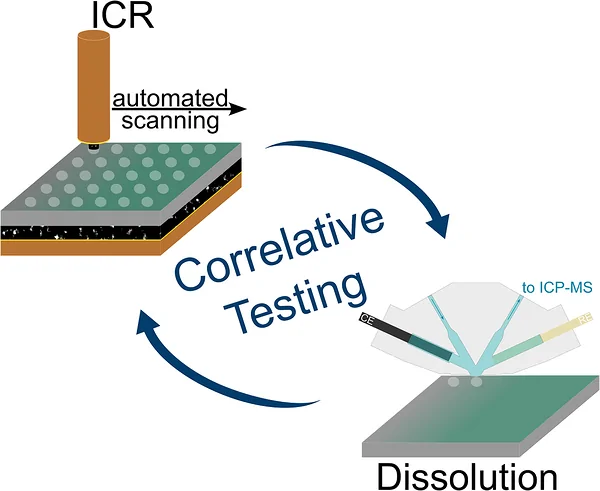

## 1 Introduction

To mitigate climate change, defossilization, with green hydrogen playing a central role, is urgently required. Green hydrogen is produced via water electrolysis powered by renewable energies. Among the available electrolysis technologies, proton exchange membrane water electrolysis (PEMWE) is particularly promising for integration with intermittent renewable electricity, owing to its rapid start‐stop capability, broad partial‐load ranges, and high achievable current densities [1, 2]. The major drawback are the high costs, hindering green hydrogen's commercial competitiveness against fossil‐derived hydrogen, primarily due to the demanding operation conditions that necessitate expensive, corrosion‐resistant materials, particularly at the anode [3, 4]. Besides iridium‐based anode catalysts, bipolar plates (BPPs) represent a major cost driver, accounting for up to 50% of the stack costs [5, 6]. BPPs separate single PEMWE cells, conduct heat and current while providing mechanical support to the stack [7]. Consequently, a corrosion‐resistant material with high electrical and thermal conductivity accompanied by low interfacial contact resistances (ICR) to the adjacent porous transport layer (PTL) is required [8]. Ti is currently the state‐of‐the‐art material, often further coated with noble metals to reduce the ICR [9, 10]. Substantial research efforts focus on replacing Ti with lower‐cost alternatives such as stainless steel [11, 12, 13, 14]. However, Ti replacement is particularly challenging at the anode side due to the corrosion of most materials under the prevailing conditions, often necessitating additional protective coatings [13, 15, 16].

Generally, the corrosion of BPPs can lead to two critical consequences: i) material dissolution [13] and ii) increased ICR caused by the formation of less conductive surface (oxide) layers [11, 14, 17]. Both phenomena potentially degrade PEMWE performance. Dissolved ions may poison the cell by blocking catalytic active sites [18], reducing membrane conductivity [19, 20, 21], or accelerating its chemical degradation [22, 23, 24, 25], while an elevated ICR introduces additional ohmic losses, thereby decreasing faradaic efficiency. Hence, evaluating novel BPP materials for PEMWE operation conditions demands assessing both their electrochemical dissolution stability *and* the ICR. Our previously introduced adjusted scanning flow cell enables the characterization of the electrochemical dissolution stability of BPP materials online during *ex situ* electrochemical testing by coupling with an inductively coupled plasma mass spectrometer (SFC‐ICP‐MS) [13, 15, 16]. However, the SFC's active area is small (mm^2^ range) compared to conventional setups used for ICR characterization, with a minimum of approximately 0.6 cm^2^ [26].

To tackle this drawback, we introduce a miniaturized ICR measurement setup with a sampling diameter of 2 mm. By mounting the setup on a movable xyz‐stage and controlling all components with a custom LabVIEW‑based software application, originally developed for electrochemical testing [27], we enable rapid, automated ICR mapping across sample surfaces. This configuration allows correlative dissolution measurements via SFC‐ICP‐MS together with occurring changes in ICR, for example, due to surface oxidation, as we demonstrate for Ti. Moreover, the setup facilitates investigation of coating thickness effects, as demonstrated for Pt‐coated Ti with a gradient in Pt thickness.

## 2 Results and Discussion

Similar to larger‐scale ICR setups, the sample is sandwiched between two gas diffusion layers (GDL, Figure 1a). Because the resistance is measured throughout the entire setup, multiple resistances are measured in series. A detailed description of the setup and required calculations for extracting the ICR of interest, including details about the error propagation, is provided in the Supporting Information (SI, Section 1). We acknowledge that the extraction of the ICR is based on several assumptions that may influence the resulting values. However, ICR values generally depend strongly on the used experimental setup, and no universal ICR value exists for a given material, as recently highlighted by Chergui et al. [28]. Nevertheless, as shown later, our setup yields ICR values in good agreement with previously reported values, and trends within a given study remain unaffected by the assumptions made for ICR extraction. For the extraction of the ICR, a calibration measurement is required in which only the small GDL attached to the Cu rod is measured (Figure S1a). A highly stable calibration value is achieved across the entire Au‐coated Cu‐plate (Figure 1b), enabling ICR mapping across the sample and demonstrating the long‐term durability of the used GDL over multiple consecutive measurements. Moreover, we have tested the reliability of the calibration for various assemblies (Figure S6). After an initial slight decrease, suspected to be caused by the compressibility of the GDL, the calibration value stabilizes and remains within the same range.

**FIGURE 1:**
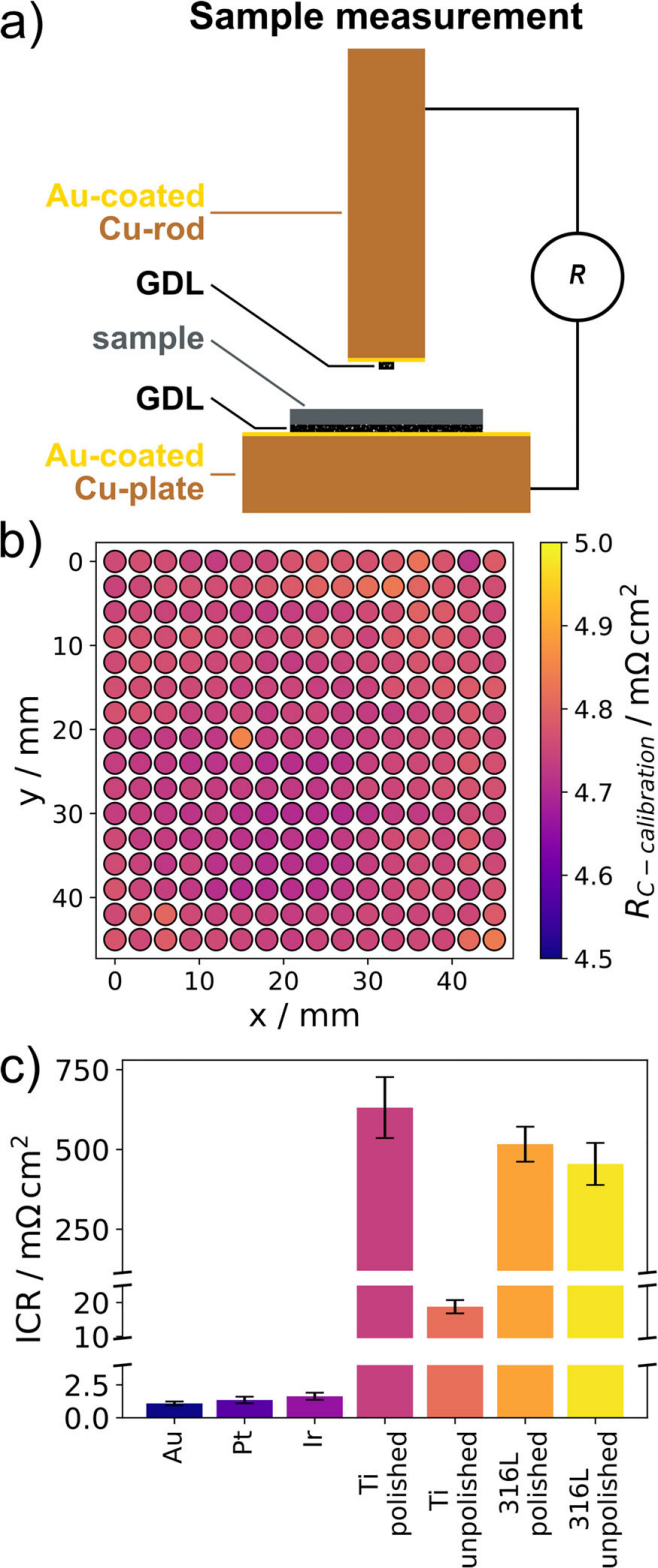
(a) Schematic drawing of the miniaturized ICR‐setup. Further details are given in the Experimental Section (SI, Section 1). (b) Calibration measurement over 256 spots (16 × 16 pattern with 3 mm x 3 mm distance, meander starting at (0|0)). Average value of 256 spots: 4.75 ± 0.02 mΩ cm^2^. (c) Average with standard deviation of ICR scanning across 49 individual spots of various pristine materials (refer to Figure S7 for single measurements). For consecutive measurements on one sample spot, refer to Figure S11.

The ICR of various pristine materials was measured by scanning across 49 locations per sample, whose average is shown in Figure 1c (Figure S7 displays the individual measurements). All noble metals exhibit low ICR values. As expected, we measure the lowest ICR value for Au, followed by Pt and Ir; however, the deviations between the materials are minor. Compared to previously reported values, we measure ICR values in a similar range for Au and Pt [17]. The ICR values of 316L and Ti are increased compared to Au, Pt, and Ir. This is an expected trend, and Pt‐ and Au‐coatings on Ti and 316L are commonly employed to lower the ICR [26, 29, 30]. 316L displays a high ICR value for both polished and unpolished (as received) conditions, while the ICR value is significantly lower for unpolished Ti compared to polished Ti, even though the samples are cut from the same Ti sheet. We assume differences in surface roughness to cause this behavior. It has already been reported for 316L that decreasing surface roughness increases the ICR [31, 32]. To further elaborate on this influence, we have tested an additional Ti sample set with varying surface roughness. We observe a similar trend for Ti: the smoother the sample surface, the higher the ICR as the actual contact area decreases (Figure S8). As the surface roughness of the unpolished and polished Ti samples used for ICR testing in Figure 1c differs (Figure S9), we attribute the significantly lower ICR of the unpolished Ti sample compared with its polished counterpart to its higher surface roughness. The miniaturized ICR setup also shows the expected decrease of the ICR with increasing compaction force (Figure S10) [11, 33, 34]. Within this study, the small GDL was pressed onto the sample with a force equaling ∼140 N cm^−2^, which is comparable to the value specified by the DOE for *ex situ* BPP testing for fuel cells [35]. This pressure also lies within the range of typical compression pressures used in PEM electrolyzers (Figure S10), for which no specific value for *ex situ* BPP testing has been established by the DOE, and is consistent with values commonly used by other researchers for ICR measurements [11, 14, 34].

For repetitive measurements on a single sample spot, we have observed different behaviors between noble and non‐noble materials. For noble materials, scanning and repetitive measurements at a single spot yield similar ICR values (averages of 49x and 50x measurements, respectively), whereas repetitive measurements on a single spot increase the ICR over time for Ti and 316L (Figure S11, Table S1–S2). As we observe random fluctuations and no systematic increase across consecutive measurements on different spots (Figure S7d–g, measurements following the order spot 1–49), we conclude that this behavior is caused by oxidation of the measurement spot during repetitive measurements and not caused by degradation of the GDL. During the measurement, a relatively high current passes through the sample (*j* ≈ 0.86 A cm^−2^), and as the GDL is porous, the local current density is even higher. This might lead to local heating [29] and oxidation of the samples, resulting in increased ICRs. However, as a significant increase is observed only after many consecutive measurements on a single spot and the difference from single measurement to single measurement is low (Figure S11c), this does not falsify the results obtained as no other measurements involving multiple consecutive measurements at the same spot were conducted in this study. Still, future studies will further elaborate on optimized measurement parameters such as applied measurement time and current to overcome this phenomena. Additionally, assembly‐to‐assembly reproducibility was evaluated for two Ti samples by performing a second ICR scan on each sample after disassembling and reassembling the experimental setup. Both scans yield a similar average ICR across the entire sample (Table S3), demonstrating good reproducibility. However, slight deviations were observed at individual measurement locations (Figure S12). These differences are attributed to the inherent measurement uncertainty and likely also to minor sample misalignment during reassembly, resulting in a small offset of the probed positions. Consequently, local surface inhomogeneities, such as scratches, may have contributed to the observed variations.

Taking together, these results show that the miniaturized ICR setup, featuring a substantially smaller sampling area (Ø = 2 mm) than conventional setups, enables localized ICR measurements, yielding results comparable to those with larger‐scale setups. In the following, we have used the miniaturized ICR setup for the following purposes: i) Complementary ICR measurements after electrochemical dissolution analysis by SFC‐ICP‐MS measurements and ii) Scanning across Ti samples with a gradient in Pt‐coating thickness.

To validate the applicability of the developed ICR setup to detect variations in the ICR value after electrochemical testing, unpolished Ti was oxidized for 600 s in 10 mM H_2_SO_4_ at increasing potentials (SFC‐ICP‐MS plots shown in Figure S13). The dissolved mass of Ti increases with applied potential; however, fluctuations increase at elevated potential (Figure 2b). Similarly, the transferred charge *Q* increases, but above 2.0 V, the average values lie within the range of errors (Figure 2b). This results in a visible step in dissolution efficiency (DE), a metric of how much of the transferred charge is consumed for dissolution (calculation details: SI, Section 4). The DE is similar for measurements between 1.4 and 1.8 V, while it decreases at applied potentials above 2.0 V. This trend may partly arise from the onset of oxygen evolution, which consumes an increasing fraction of the applied charge. However, the lower DE also hints towards enhanced surface passivation and the formation of a more protective oxide layer at higher potential. Visual differences in the measurement spots appear depending on the applied potential (Figure 2a). At a potential of 2.0 V, the spots become more visible, and they color further after measurements at potentials of 2.6 V and higher, again indicating higher passivation at elevated potential.

**FIGURE 2:**
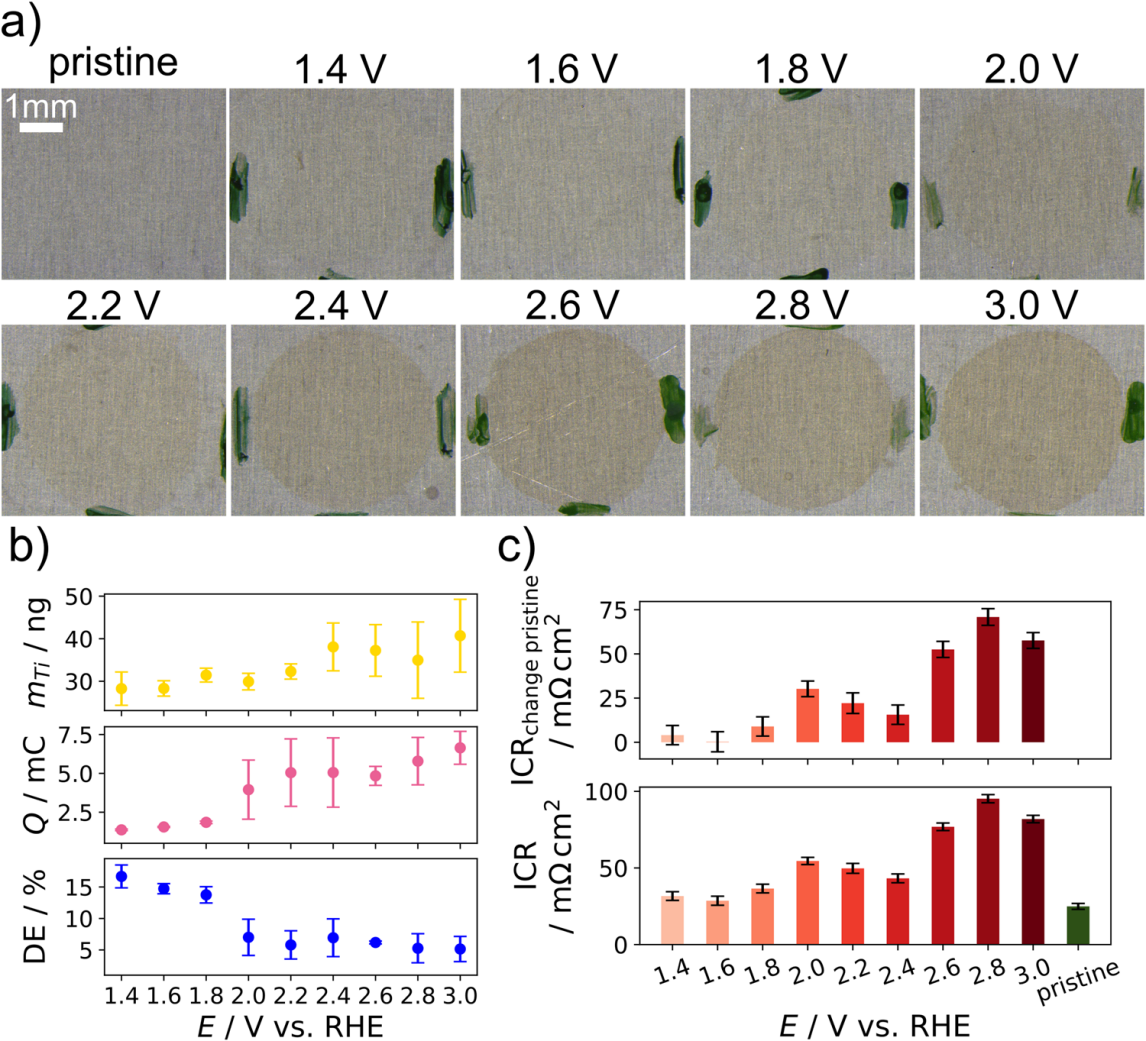
SFC‐ICP‐MS and correlative ICR measurements of Ti after oxidation at various potentials for 600 s in 10 mM H_2_SO_4_ at *T_stage_* = 60°C. (a) Light microscope images of unpolished, pristine Ti and of the measurement spots after oxidation. Scale bar applies to all images. (b) Analysis of SFC‐ICP‐MS measurements displayed in Figure S13: dissolved mass *m_Ti_* and transferred charge *Q* during the measurement as well as the dissolution efficiency DE (average and standard deviation of three measurements on independent sample locations per potential, refer to Figure S15 for individual measurements), (c) ICR measurements showing the ICR change compared to its pristine value (top) and the absolute ICR (bottom) depending on the applied potential (average of three measurements per applied potential, refer to Figure S15 for individual measurements).

After electrochemical testing, the ICR change of Ti was measured using the miniaturized ICR setup, revealing distinct differences depending on the applied potentials (Figure 2c). For potentials applied between 1.4–1.8 V in average, no ICR increase compared to the pristine state is detected. An increase around 15–30 mΩ cm^2^ is detected for measurements between 2–2.6 V, and the ICR increases even more for potentials above 2.6 V (+50–70 mΩ cm^2^). We acknowledge that, particularly at the lower potentials, the ICR values for individual spots exhibit considerable scatter (Figure S15). This variability likely stems from a combination of factors, including oxidation kinetics, alterations in surface roughness, localized sample inhomogeneities such as scratches, and the intrinsic measurement error. The precision of the ICR measurement could be further enhanced by coating the sample's backside with a noble metal. As the sample is sandwiched between two GDLs, but only one side is corroded, the reported ICR must be corrected by subtracting the backside contribution (SI, Section 1.1). This correction introduces additional uncertainty, especially when both the ICR and its standard deviation for the backside are large.

Nevertheless, we can successfully detect changes in the ICR after electrochemical testing and observe a clear potential dependence of the ICR. A greater increase in the ICR with increasing potential has also been reported by Laedre et al [17]., though for a different material (TaITO‐coated Ti). A linear increase in Ti oxide thickness with increasing potential has previously been reported for oxidation in 0.1 M H_2_SO_4_ [36]. Similarly, an increase in oxide growth was also detected for dynamic polarization in 0.05 M H_2_SO_4_ [37] and in 1 M  H_2_SO_4_ [38]. Therefore, we suspect that a thicker oxide layer grows on Ti at higher potential, increasing the ICR value, which aligns with the reduced DE and the changes in coloration observed. This shows that the miniaturized setup is capable of complementary dissolution and ICR measurements.

To meet the ICR targets for BPPs (<10 mΩ cm^2^_,_ as specified by the DOE for fuel cells; no specification available for PEMWE [35, 39]), Ti must be coated, for example, with noble metals, as already reported in literature, to reduce the ICR [30, 40, 41, 42]. Most of the Pt coatings employed on Ti are relatively thick (several hundreds of nm to µm) [30, 40, 41, 42]. However, the right balance must be found: a thick enough Pt layer to minimize the ICR while avoiding overly thick layers that increase the cost and prevent PEMWE from meeting its total platinum group metal content targets [39] (Figure S16). To explore this, we deposited two wedge‐type Pt‑thickness gradients on Ti: ∼0–33 nm_Pt_ and ∼10–80 nm_Pt_ across 80 mm length (Figure S17).

Figure 3 displays the ICR measurements across the Pt wedge, ranging from 0–33 ± 1.8 nm_Pt_ on Ti. The ICR is increased at the thin end of the Pt coating (around 7–8 mΩ cm^2^), but it quickly drops when the Pt thickness increases (Figure 3c,d). The elevated ICR value measured at ∼0 nm_Pt_ is lower than the pristine Ti ICR value (Figure 1c, unpolished Ti). We attribute this to the additional Ti adhesion layer. During the deposition when highly‐pure Ti is deposited onto the native TiO_2_ layer on the Ti substrate, interfacial reduction may occur, leading to the formation of substoichiometric TiO_x_, which can be more electrically conductive than stoichiometric TiO_2_ [43]. The sharp ICR decrease occurs in the same range as the Pt coating on the sample becomes visible on Ti (Figure 3a). Then, even though the Pt thickness on Ti increases further, the ICR value remains within a stable range of 1.1 ± 0.3 mΩ cm^2^. Using a piecewise linear regression approach to analyze the ICR profile, we determined that the transition to this stable ICR regime corresponds to a Pt thickness of approximately 3.8 ± 1.4 nm_Pt_ (Figure 3b, see Figure S19–S22 for further details).

**FIGURE 3:**
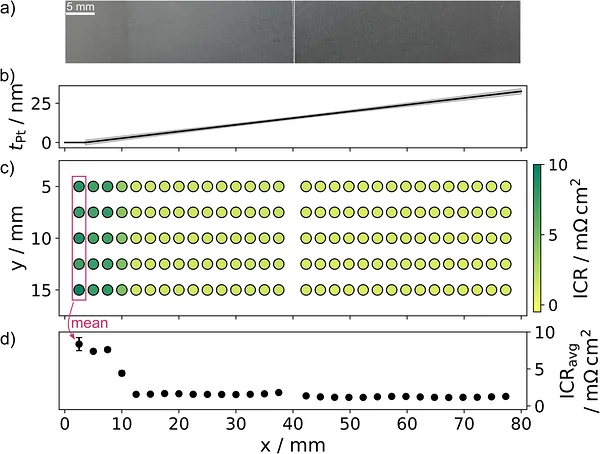
ICR measurements (sampling diameter: 2 mm) across Pt‐coated Ti. Topside: ∼0–33 ± 1.8 nm_Pt_, backside: 16 ± 2.8 nm Pt coating. (a) Micrographs of the Pt‐wedge across the sample. (b) Linear fit with uncertainty of Pt‐thickness gradient across sample (refer to SI Section 5 for further details). (c) ICR measurements across the sample at different locations and d) average with standard deviation of 5 measurements at a location with identical Pt‐thickness of c).

Increasing the thickness of the wedge, ranging from ∼10–80 nm_Pt_ across Ti, did not result in any further significant decrease in ICR value (Figure S23), resulting in an average ICR of 0.7 ± 0.2 mΩ cm^2^. Even though this average value is slightly lower than the measured value in the stable range of the 0–33 ± 1.8 nm_Pt_ wedge, no systematic decrease along the wedge is detected for both samples. Therefore, we assume that these differences are rather caused by measurement inaccuracies than actual improvement in the ICR value.

Based on our results, we conclude that a very thin Pt layer on Ti is sufficient to significantly improve the ICR value. Theoretical calculations using the ICR values measured for uncoated Ti and Pt‐coated Ti reveal that already at a Pt‐coverage of 50%, the effective ICR would be drastically reduced (Figure S24). Pt is known to form a continuous film already above a nominal thickness of approximately 1–2 nm, corresponding to the transition from isolated Pt islands to a continuous Pt layer [44]. However, transmission electron microscope analysis of sputtered Pt on Si_3_N_4_ revealed a surface coverage of approximately 85 % for a 4.7 nm Pt layer (Figure S25). This demonstrates that the coating thickness required to form a continuous Pt layer depends on the substrate. Nevertheless, considering the ICR results and the theoretical analysis of the influence of Pt surface coverage on the effective ICR (Figure S24), we assume that a high surface coverage was achieved for Pt thicknesses above 3.8 ± 1.4 nm_Pt_ on the present Ti surface. This interpretation is consistent with the onset of a visible Pt coating and is further supported by the X‐ray fluorescence (XRF) measurements (Figure S22). While a thicker Pt coating on Ti might enhance in‐plane conductivity, this does not beneficially influence the ICR, as also the theoretical ICR considerations have demonstrated (Figure S24). We acknowledge that the critical threshold of Pt thickness may differ for other Ti samples or materials. For example, full Pt coverage will depend on the sample's surface roughness, which can affect the ICR values measured. However, our reported values are in good agreement with a recently reported threshold of 5 nm for a Pt layer on TiO‐coated 316L where increasing the Pt thickness to 20 nm resulted in no further improvement in the ICR [45]. Similar trends are also reported for PTLs. For pristine samples, Pt‐coated Ti PTLs with Pt coating thicknesses of 6, 11, and 17 nm exhibited similar ICR values, while the ICR of the thinnest Pt coating was significantly enhanced after electrochemical testing attributed to coating detachment [46]. At the cell level, an 8 nm Pt‐coating on a Ti‐PTL has been shown to provide similar performance and durability comparable to those of thicker coatings up to 50 nm_Pt_ [47]. Therefore, we assume that, in general, only a very thin layer of noble metal is required to improve the ICR drastically. However, the exact threshold thickness will depend on the substrate material, its surface condition and roughness as well as the Pt coating quality.

As these Pt layers are very thin, the question arises whether they can maintain a low ICR during electrochemical operation. While we assume that such a thin layer might withstand the conditions expected at the BPP of a PEMWE [48], Pt coatings are now also being applied to the anode catalyst‐facing side of the PTL to reduce the interface ICR [46, 47, 49, 50]. A recent study has shown that Pt tends to dissolve and migrate into the membrane, leading to degradation and safety concerns [51]. Therefore, in future work, we will utilize the workflow we presented to test dissolution stability and ICR correlatively to assess the performance of Pt layers with varying thickness under different electrochemical conditions expected within the anode side of PEMWE systems.

## 3 Summary and Conclusion

In this study, we introduced a correlative workflow for dissolution and ICR measurements by developing a miniaturized ICR measurement setup with a 2 mm sampling diameter. We demonstrated the capabilities of this setup by using Ti, for which the ICR increase after electrochemical testing strongly depends on the applied potential. Significant ICR increases were observed at potentials above 2.0 V and 2.6 V, respectively. Furthermore, measuring the local ICR across a Pt‐coated Ti sample with a linear gradient in Pt thickness (∼0–33 nm_Pt_) revealed that even a very thin Pt layer (3.8 ± 1.4 nm_Pt_) drastically improves the ICR for the given Ti substrate. Thicker Pt layers (up to ∼80 nm_Pt_) do not significantly improve the ICR. The electrochemical stability testing of Pt‐wedge samples replicating different conditions on the anode side of a PEMWE, with subsequent ICR testing, is an important subject to a future study. Such investigations could provide further insight into whether thicker Pt coatings are required to maintain a low ICR after oxidation of the underlying Ti substrate. In addition, the setup could be used to map the ICR of larger samples after uniform corrosion testing, enabling the detection of localized corrosion phenomena (e.g., pitting corrosion) that result in an inhomogeneous ICR distribution. This could be particularly valuable for investigating BPPs after operation in an electrolyzer, where spatial variations in ICR between channels and lands may provide information on the influence of local operating conditions and corrosion processes. For example, different iridium dissolution behaviors have been already observed under lands and channels [52, 53]. Depending on the geometry of the BPP, however, adjustments to the ICR setup to accommodate larger samples, together with further miniaturization of the probe, may be required to enable ICR measurements on real BPP geometries. In summary, the presented workflow lays the foundation for the highthroughput correlative stability and ICR testing of novel PEMWE materials, as the setup can detect differences in dissolution and ICR across samples with compositional changes (Figure 4). This paves the way for accelerated material testing to identify more costeffective components for PEMWE.

**FIGURE 4:**
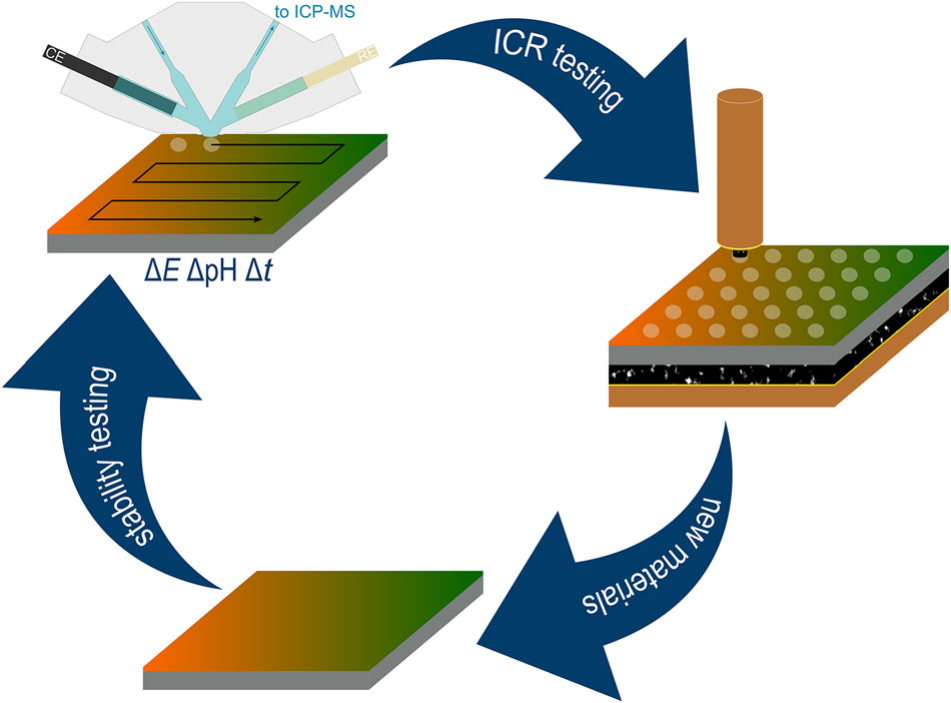
Outlook of the developed miniaturized ICR setup enabling correlative dissolution stability measurements by SFC‐ICP‐MS and subsequent ICR characterization for the identification of novel cost‐effective materials.

## Author Contributions

**Lena Fiedler**: conceptualization (equal), investigation (lead), methodology (lead), formal analysis (lead), visualization (lead), software (lead), writing – original draft (lead). **Andreas Körner**: software (supportive). **George Pätzold**: investigation (supportive), formal analysis (supportive). **Elaheh Akbarnejad**: investigation (supportive), formal analysis (supportive). **Alan Savan**: investigation (supportive), formal analysis (supportive). **Simon Thiele**: resources (equal), funding acquisition (supportive). **Dominik Dworschak**: resources (equal), data curation (lead). **Alfred Ludwig**: supervision (equal), resources (equal). **Karl J. J. Mayrhofer**: supervision (equal), conceptualization (equal), funding acquisition (lead), resources (equal). **Andreas Hutzler**: conceptualization (equal), supervision (equal), project administration (lead). **All authors** contributed to the Writing – Review & Editing (equal).

## Generative AI Statement

ChatGPT (OpenAI) was used to improve the readability of the manuscript and to provide coding support. After using this tool, the authors reviewed and edited the content as needed and take full responsibility for the content of the published article.

## Conflicts of Interest

The authors declare no conflicts of interest.

## Supporting information




**Supporting File**: smtd71010‐sup‐0001‐SuppMat.pdf.
The authors have cited additional references within the Supporting Information [54, 55, 56, 57, 58, 59, 60, 61, 62, 63, 64].

## Data Availability

The data that support the findings of this study are available from the corresponding author upon reasonable request.
