# Supplementary material for: Towards High‐Throughput Testing of Bipolar Plate Materials for Proton Exchange Membrane Water Electrolyzers: Correlative Contact Resistance and Stability Analysis
Source: Small Methods. 2026 Sep 3;10(19):e71010. doi: 10.1002/smtd.71010 (PMC13646020; doi:10.1002/smtd.71010)
Supplement: Supplementary file 1 [file SMTD-10-e71010-s001.pdf]

Supporting Information

Towards High-Throughput Testing of Bipolar Plate Materials for Proton Exchange Membrane Water Electrolyzers: Correlative Contact Resistance and Stability Analysis

Lena Fiedler,*[a,b] Andreas Körner,[a,b] George Pätzold,[a,b] Elaheh Akbarnejad,[c] Alan Savan,[c] Simon Thiele,[a] Dominik Dworschak,[a] Alfred Ludwig,[c] Karl J. J. Mayrhofer,[a,b] and Andreas Hutzler*[a]

[a] L. Fiedler, A. Körner, G. Pätzold, Prof. Dr. S. Thiele, Dr. D. Dworschak, Prof. Dr. K. J. J. Mayrhofer, Dr. A. Hutzler
Helmholtz Institute Erlangen-Nürnberg for Renewable Energy (IET-2)
Forschungszentrum Jülich GmbH
Cauerstraße 1, 91058 Erlangen, Germany
E-mail: l.fiedler@fz-juelich.de, a.hutzler@fz-juelich.de

[b] L. Fiedler, A. Körner, G. Pätzold, Prof. Dr. S. Thiele, Prof. Dr. K. J. J. Mayrhofer
Department of Chemical and Biological Engineering
Friedrich-Alexander-Universität Erlangen-Nürnberg
Egerlandstraße 3, 91058 Erlangen, Germany

[c] Dr. E. Akbarnejad, A. Savan, Prof. Dr. A. Ludwig
Materials Discovery and Interfaces
Institute for Materials
Ruhr-Universität Bochum
Universitätsstr. 150, 44801 Bochum, Germany

1 Experimental

1.1 Miniaturized ICR-Setup

A Cu-rod (Ø = 10 mm) and Cu-plate (50 x 50 mm), both purchased from Hans-Erich-Gemmel & Co GmbH, were ground (SiC, from P240 to P4000, Struers) and polished (3 µm diamond suspension, Mol R3, Struers) before sputter-coating with Au (Cressington 108). A carbon GDL (Ø = 2 mm, TGP-H-120, Toray) was attached to the Au-coated Cu-rod. This rod-assembly was attached to a force sensor and pressed with a force of 4.4 N (~140 N cm^{-2}) on the sample for all measurements, unless otherwise stated. Below the rod-assembly, the Au-coated Cu-plate is placed on a movable xyz-stage, facilitating scanning across multiple measurement spots on the sample. For the calibration measurement, the rod assembly was pressed solely onto the Au-coated Cu plate. In contrast, for sample measurements, an additional carbon GDL (same dimensions as the sample, TGP-H120, Toray) was placed below the sample on the Au-coated Cu plate. A scheme of the setup is displayed in Figure S1.
If the ICR was measured after electrochemical measurement, the ICR was measured in the center of the corroded area by using a top view camera with known offset for accurate positioning of the small GDL.

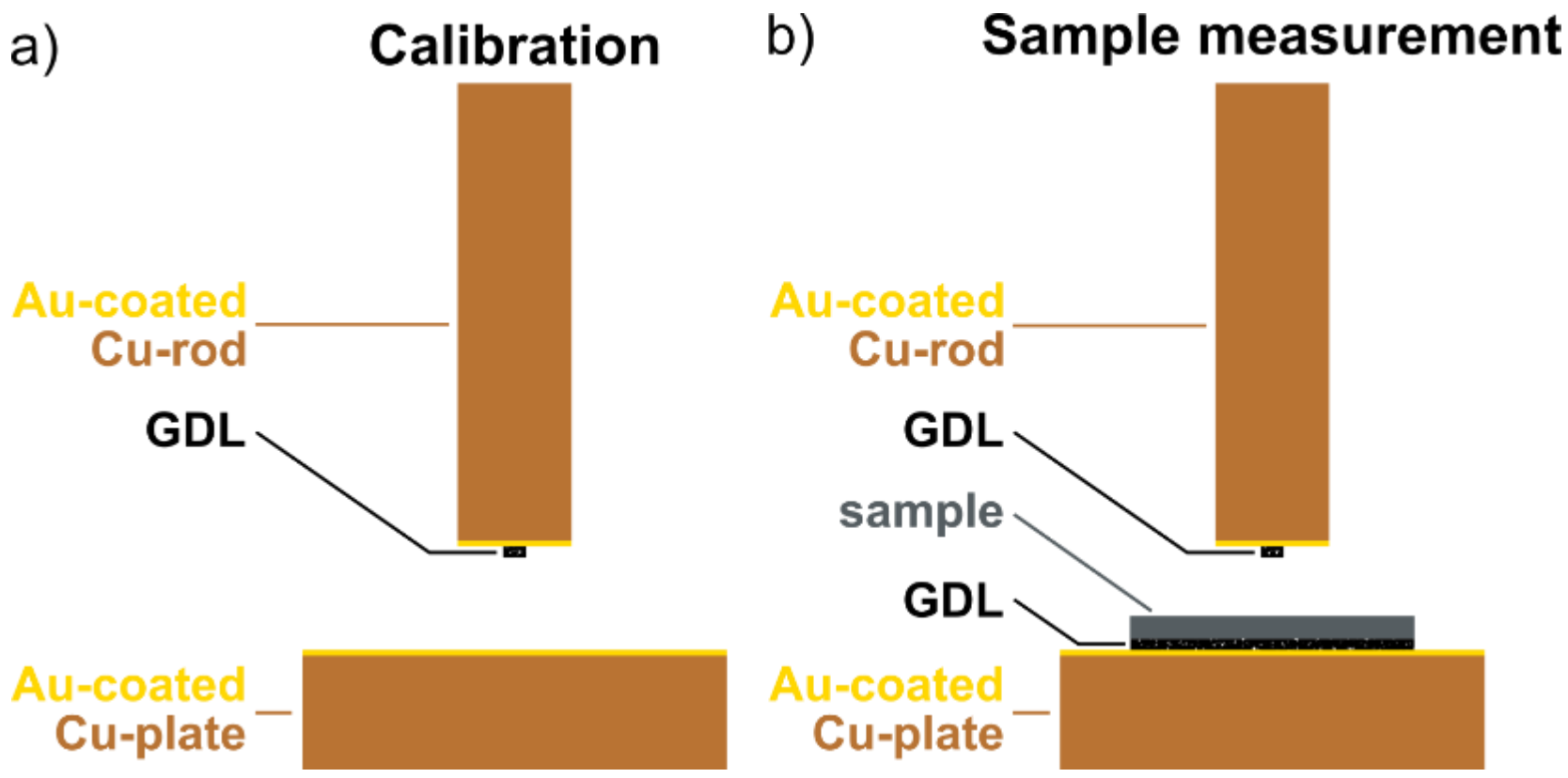

Figure S1. Scheme of the miniaturized ICR measurement setup. a) Calibration measurement and b) sample measurement.

All measurements were performed using a potentiostat (Reference 620, Gamry Instruments) in galvanostatic mode in a two-electrode configuration over 20 s per individual measurement. The resistance was calculated by Ohm's law (Equation (1)). However, the measured resistance during pristine sample measurements R_1 is composed of multiple resistances: the bulk resistances of the sample ($R_{B,\ sample}$), the PTL ($R_{B\ ,\ GDL}$), and Cu parts ($R_{B,\ Cu}$) as well as the interfacial contact resistances between Au-coated Cu plate/rod and GDL ($R_{ICR,\ Cu\text{-}GDL}$) and between the sample and GDL ($R_{ICR,\ sample}$), and the resistance of the circuit ($R_{circuit}$) (Equation (2)). To extract the ICR of the sample, the calibration measurement (R_2) is done with only the small GDL placed between the Cu parts (Equation (3)). With the assumption that the bulk resistances are negligible, the subtraction of the two resistances and division by two yields the ICR of the sample (Equation (4)).[1]
As only one side of the sample is corroded, the measured resistance for corroded samples (R_3) includes once the ICR between the pristine sample and GDL ($R_{ICR,\ sample}$) and once between the corroded sample and GDL ($R_{ICR,\ sample,\ corroded}$) (equation (5)). Therefore, the ICR of corroded samples is calculated by subtracting the calibration and the ICR of the pristine sample from the measured value (Equation (6)). Subtracting the ICR between the pristine sample and GDL ($R_{ICR,\ sample}$) twice, yields the change in ICR value caused by corrosion relative to its pristine state (equation (7)).
For the samples with a gradient in Pt-thickness, the measured ICR includes once the ICR between the Pt-wedge and GDL ($R_{ICR,\ Pt\text{-}wedge}$) as well as the ICR of the homogenously coated backside and GDL ($R_{ICR,\ backside}$) (Equation (8)). To extract the ICR between the Pt-wedge and GDL, and thereby the influence of the Pt layer thickness on the ICR, the calibration and the ICR of the backside must be subtracted from the measured value (Equation (9)).

$$R = \frac{U}{I} \tag{1}$$

$$R_1 = 2\,R_{ICR,\ sample,} + 2\,R_{ICR,\ Cu-GDL} + R_{circuit} + 2\,R_{B,Sample} + 2\,R_{B,GDL} + 2\,R_{B,Cu} \tag{2}$$

$$R_2 = 2\,R_{ICR,\ Cu-GDL} + R_{circuit} + R_{B,GDL} + 2\,R_{B,Cu} \tag{3}$$

$$R_{ICR,\ sample} = \left(\frac{R_1 - R_2}{2}\right) \tag{4}$$

$$R_3 = R_{ICR,\ sample} + R_{ICR,\ sample,\ corroded} + 2\,R_{ICR,\ Cu-GDL} + R_{circuit} + 2\,R_{B,Sample} + R_{B,GDL} + 2\,R_{B,Cu} \tag{5}$$

$$R_{ICR,\ sample,\ corroded} = R_3 - R_2 - R_{ICR,\ sample} \tag{6}$$

$$R_{ICR,\ sample,\ corroded,\ change\ pristine} = R_3 - R_2 - 2 \cdot R_{ICR,\ sample} \tag{7}$$

$$R_4 = R_{ICR,\ Pt-wedge} + R_{ICR,\ backside} + 2\,R_{ICR,\ Cu-GDL} + R_{circuit} + 2\,R_{B,Sample} + 2\,R_{B,GDL} + 2\,R_{B,Cu} \tag{8}$$

$$R_{ICR,\ Pt-wedge} = R_4 - R_2 - R_{ICR,\ backside} \tag{9}$$

To report the area-specific contact resistance in Ω cm^2, the calculated resistance is multiplied by the sampling area *A* (3.14 mm^2).[1]

We acknowledge that the extraction of the ICR is based on several assumptions. Compared to most conventional setups described in the literature, the main difference is that our setup employs two GDLs of different sizes. To investigate the influence, we have done a control experiment measuring the resistance between two small GDLs and between a large GDL (25x25 mm) and a small GDL (Figure S2a). Owing to geometric constraints of the experimental setup, a calibration using only the large GDL was not feasible, as the larger dimensions of the Au-coated Cu rod would result in a different contact area. As both measurements yield a similar resistance (Figure S2b), this can justify the assumptions that the larger GDL-interfaces does not significantly alter the measured value. The higher value compared the standard ICR measurement (Figure S6) is attributed to the additional contact resistance between the two sandwiched GDLs.

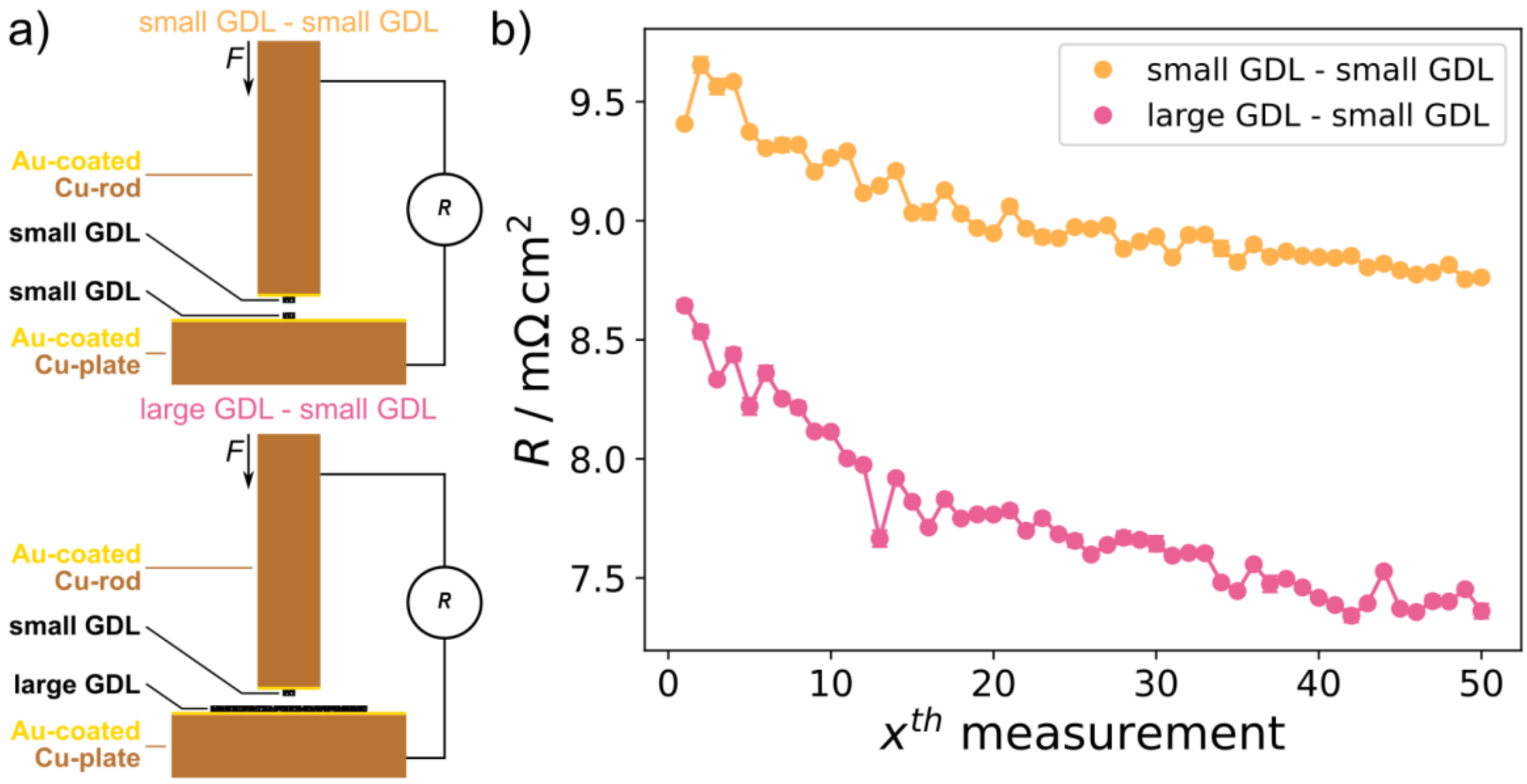

Figure S2. Influence of GDL geometry on measured resistance. a) Schematic drawing showing the two measurements: once the resistance is measured of two small GDLs and once the resistance is measured if a small and a large GDL are compressed. b) Measured resistance for 50x consecutive measurements.

1.1.1 Error Analysis

Equation (9) has been used to calculate the sample standard deviation σ of the mean $\bar{x}$ calculated from multiple individual values x_i.

$$\sigma = \sqrt{\frac{1}{N-1}\sum(x_i - \bar{x})^2} \tag{10}$$

When a mean value z was calculated from individual values z_i, each of which already has a standard deviation, two standard deviations $\sigma_{mean,1}$ and $\sigma_{mean,2}$ were calculated to determine the standard deviation of z (Equations (11) and (12), respectively). The larger of the two values is reported as the standard

deviation of z. This approach was, for example, applied when measurements obtained at different positions across a sample were averaged.

$$\sigma_{mean,1} = \sqrt{\frac{1}{N-1}\sum(z_i - \bar{z})^2} \tag{11}$$

$$\sigma_{mean,2} = \sqrt{\frac{\sum(\sigma_{z_i})^2}{N-1}} \tag{12}$$

If values were calculated by adding or subtracting average values, their standard deviations were summed up to report the standard deviation of the calculated value r (Equation (13)). This conservative approach accounts for potential correlations between the individual uncertainties, which cannot be fully excluded in the present measurements. This approach has been used when the ICR value of samples was calculated (Equations (4), (6), (7), and (9)). It accounts for the calibration error and, in the case of corroded samples, the error associated with the pristine measurement, as well as the error associated with the ICR of the backside measurement in the case of the Pt wedge sample.

$$\begin{aligned} r &= x \pm y \\ \sigma_r &= \sigma_x + \sigma_y \end{aligned} \tag{13}$$

1.1.2 Current-Voltage Linearity

Figure S3 displays a sweep over different applied currents for the calibration measurement. As expected, the voltage and current density exhibit a linear relationship over the applied range. A linear fit was used to determine the resistance of the calibration setup, yielding a similar value, as the galvanostatic measurement performed as standard at an applied current density of 0.86 A cm^{-2} (4.5 mΩ cm^2). We have actively decided to apply a relatively low current of 0.027 A, for all measurements, as this already corresponds to a current density of 0.86 A cm^{-2} due to the low sampling area. There is no general current density at which the ICR is tested, but the value lies within the same range as used by other researchers.[2-14] Please note, that in some cases the sample size is not explicitly mentioned; if this was the case, the sample size given in the experimental section was used to calculate the used current density.

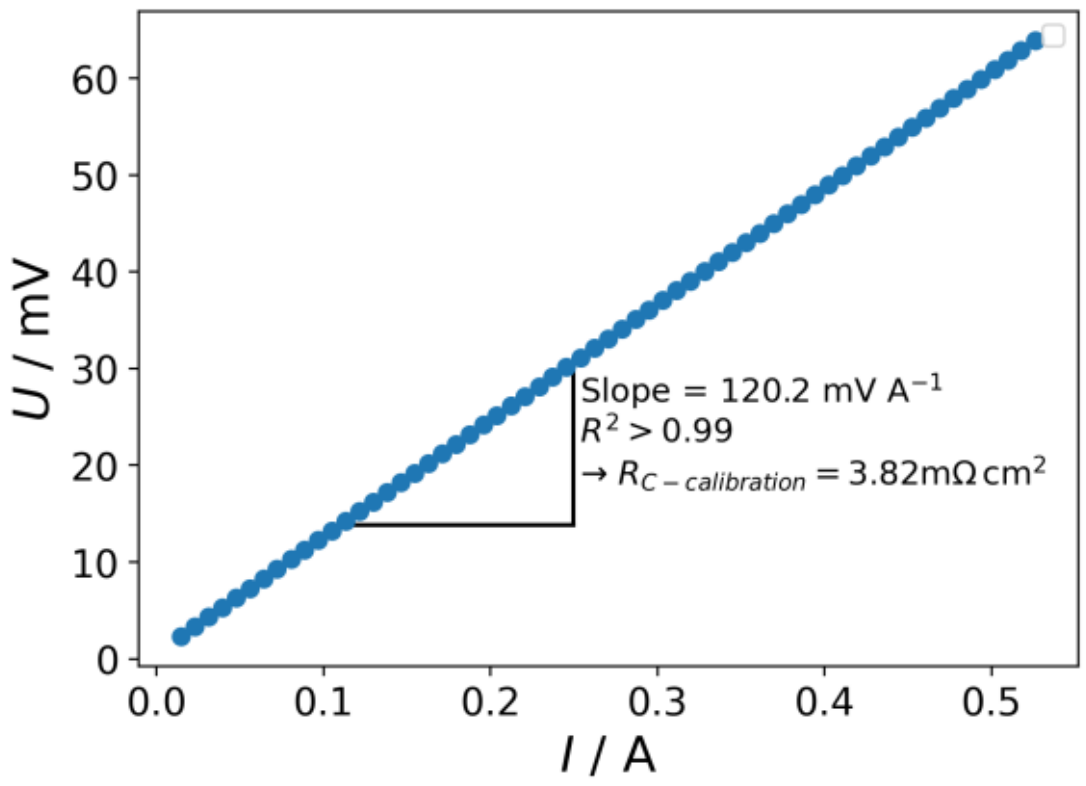

Figure S3. Linear sweep amperometry of the calibration measurement.

1.1.3 Measurement Drift and Accuracy

Figure S4 exemplarily displays the raw voltage data recorded over time during the galvanostatic ICR measurements. For the evaluation the two first measurement points were excluded, as the initial jump is attributed to an artefact of the potentiostat. As the measurement compresses the porous material, a slight drift in the measured voltage is observed. This drift can be attributed to the gradual compression of the material during the measurement, which increases the real contact area and consequently decreases the resistance. Nevertheless, the observed drift is relatively small. For the evaluation, the average voltage was determined, and its standard deviation was included in the error propagation.

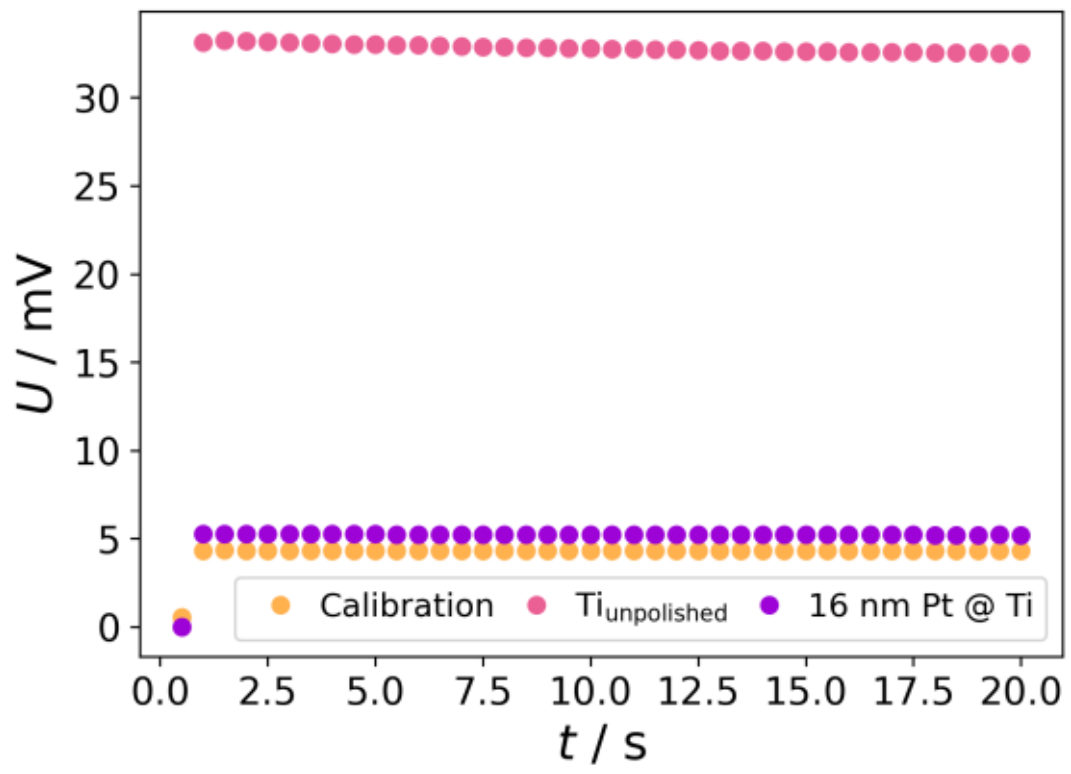

Figure S4. Measured potential over time during galvanostatic ICR measurement (I = 0.027 A) of the calibration, unpolished Ti (Evek) and the Pt-coated Ti reference sample (16 ± 2.8 nm_{Pt} on both sides). For evaluation, the first two measurement points were discarded.

The instrumental resolution of the used potentiostat is specified for the applied current and measured potential as given in Equations (14) and (15), respectively. For the standard measurement conditions this results in an accuracy of 0.31% of the applied current (27 ± 0.084 mA). The accuracy of the measured potential depends on the measured potential itself. For the measurement conditions used here (27 mA applied current and a contact area of 0.031 cm^2), the constant ±1 mV contribution to the potential accuracy corresponds to an uncertainty of approximately ±1.16 Ω cm^2 when expressed as an area-normalized resistance. This value should therefore not be interpreted as a general uncertainty applicable to all ICR measurements. The resistance measured directly across the complete setup is generally higher than the ICR values reported, except for the calibration measurements, because the ICR values are extracted from the measured resistance using the calculations described in Section 1.1.

$$I_{Applied\ Accuracy} = \pm\, 10\, pA \pm 0.05\%\, range_{\frac{I}{E}} \pm 0.2\%\, I_{applied} \tag{14}$$

$$E_{Measured\ Accuracy} = \pm\, 1\, mV\ \pm 0.2\%\, E_{measured} \tag{15}$$

1.2 Samples and Materials

Iridium (99.9%, 25 x 25 mm, t = 0.25 mm, MaTecK), platinum (99.99%, 25 x 25 mm, t = 0.25 mm, MaTecK), and gold (99.95%, 25 x 25 mm, t = 0.5 mm, ThermoFisher Scientific) were ground (SiC #500 to #4000, Struers) and polished (Mol R3 and Nap ¼, Struers) on both sides. Titanium (grade 2, 25 x 25 mm, t = 0.5 mm, Evek GmbH) and stainless steel (grade 316L, 25 x 25 mm, t = 0.5 mm, Evek GmbH) were ground (SiC #220 to #4000, Struers) and polished (Mol B3 and Nap ¼, Struers) on both sides using a semi-automatic polishing machine (Struers, LapoPol-30 with LaboForce 100). Besides, the samples were used in their pristine state as received (referred to as unpolished).

To test the influence of the surface roughness on the ICR value, titanium (25x25 mm, t = 1.5 mm, Hans-Erich Gemmel & Co. GmbH) was ground (SiC #220 to #4000, Struers) and polished (Mol B3 and Nap ¼, Struers) on both sides using a semi-automatic polishing machine (Struers, LapoPol-30 with LaboForce 100). For two samples, the grinding was stopped after SiC #220 and SiC #2000, respectively.

All samples were cleaned with a soap solution and ultrasonicated in isopropanol and DI water.

1.3 Electrochemical Stability Measurements (SFC-ICP-MS)

All electrochemical measurements were conducted using a scanning flow cell coupled on-line to an inductively coupled plasma mass spectrometer (SFC-ICP-MS), which enables time-resolved detection of dissolved species from the sample into the electrolyte. A detailed description of the setup can be found elsewhere.[15] Briefly, the electrolyte is continuously pumped through the V-shaped channels of the SFC (Ø = 3.5 mm) and fed into an ICP-MS (flow rate measured daily). At the channel's apex, the SFC has an elliptical opening facilitating contact to the working electrode (WE). A sealing ring ($Ø_{i.d.}$ = 4 mm, $Ø_{o.d.}$ = 6 mm) to seal the active area was punched from EPDM rubber sheets (1/64″ thickness, McMaster-Carr). The active area (A = 12.6 mm^2) was calculated based on the sealing's inner diameter.

A Glassy carbon rod (Ø = 1.6 mm, HTW Hochtemperatur-Werkstoffe) placed at the inlet and a leakage-free Ag|AgCl-reference electrode (LF-1.6-48, Ø = 1.6 mm, Innovative Instruments, Inc.) placed at the outlet served as counter (CE) and reference electrode (RE), respectively. As the RE is leakage-free, we exclude any corrosion enhancing influence of Cl^-. All potentials are reported versus the reversible hydrogen electrode (RHE). Therefore, the RE potential was measured daily by measuring its open circuit potential in hydrogen-saturated electrolyte versus a Pt-wire (Ø = 0.5 mm, 99.99%, MaTeck). A potentiostat (Reference 620, Gamry Instruments) was used, and the sample was placed on a temperature-controlled stage to heat the sample (T_{stage} = 60 °C). O_2-purged 10 mM H_2SO_4 (Ultrex® II, J.T.Baker, diluted with ultrapure water (18.2 MΩ cm^{-1})) was used as electrolyte. The sample was oxidized by applying various potentials (1.4, 1.6, 1.8, 2.0, 2.2, 2.4, 2.6, 2.8, and 3.0 V) for 600 s. Per potential, the measurement was repeated three times on independent locations on the samples.

Prior to entering the ICP-MS (Agilent 7900, Agilent Technologies), the downstream electrolyte was mixed with an internal standard solution (^{45}Sc for ^{47}Ti in 5% HNO_3 (CarlRoth, Ultrapur) prepared from Certipur ICP-MS standard, Merck). Daily calibration of the ICP-MS was performed using standard solutions of all analytes (Certipur ICP-MS standard, Merck). All ICP-MS measurements were performed in the time-resolved analysis mode with general purpose mode.

The dissolved mass m_{Ti} and transferred charge Q during the measurements was obtained by integration of the dissolution rate and current profile over the entire measurement time.

1.4 Surface area roughness

The surface area roughness of the materials was measured by laser profilometry with a confocal microscope (VK-X200, Keyence) at 50x magnification. The obtained height profiles were background-corrected prior to determining the surface area roughness in three independent areas per sample.

1.5 Microscopic images

Light microscopic images were taken with a conventional light microscope (M125 C with MC170 HD, Leica).

1.6 Fabrication of Platinum Coating by Sputter Deposition

Pt thin films were deposited on Ti sheets (grade 2, t = 0.5 mm, Evek GmbH) using a magnetron sputter system (DCA Instruments, Finland) in a UHV chamber where cathodes taking 100 mm diameter targets can be sequentially positioned directly above the substrate i.e. sputter-down orientation, with a target to substrate distance of 13.8 cm. To improve the adhesion of Pt, a 20 nm Ti interlayer was deposited first. To accurately determine the thickness of the deposited thin films, coatings were simultaneously deposited on photolithographically patterned Si wafers.

The Ti (99.99%, EvoChem) adhesion layer was deposited at room temperature at an Ar pressure of 0.67 Pa with a sputter rate of 0.054 nm_{Ti} sec^{-1}. Pt (99.99%, MaTeck) was deposited immediately thereafter, also at room temperature at an Ar pressure of 0.67 Pa and a sputter rate of 0.14 nm_{Pt} sec^{-1}. To create a thickness gradient across the Ti sheets, a moving shutter was used during the Pt deposition. By this, two desired wedge type layers of Pt with approximate thicknesses of 0 – 33 nm_{Pt} and 10 – 83 nm_{Pt} across the Ti sample (total length: 80 mm, with two 40 mm long samples placed next to each other) were obtained. Additionally, a reference sample with homogenous 16 nm Pt was deposited. For ICR testing, the backside of all samples was coated with a 20 nm Ti interlayer and a homogeneous 16 nm Pt coating. Figure S17 shows an overview of the samples prepared.

1.7 Profilometry measurements

To determine the thickness of the deposited films, the photolithographic lift-off pattern was removed after deposition using ultrasonication in acetone followed by isopropanol for 15 minutes each, leaving sharp steps from the film surface to the substrate. Then, profilometry measurements were conducted using a profilometer (Ambios XP-2), that was calibrated by a NIST-traceable thickness standard (step height = 9841 Å). At least six measurements were taken at each measurement spot to obtain an average value.

1.8 XRF measurements

For verification of the Pt-gradient an X-ray fluorescence spectrometer (XRF, M4 Tornado, Bruker USA) with a spot diameter of 20 µm was used. Each spot was measured with a rhodium X-ray tube and the following operating parameters: 50 kV, 600 µA, measurement time per spot: 1 s. For quantification the Pt peaks were integrated and referred to as Pt XRF intensity in this work. Each sample was measured at 101 points in direction of the gradient (X-direction) over the entire sample length. Perpendicular to the gradient (Y-direction), 10 measurements were conducted at 1 mm intervals for each x-position, yielding 1010 measured points per sample and 2020 over the entire gradient length.
A sample set without backside coating was used to avoid any influence on the measurements. The wedge-type Pt layers were deposited simultaneously, with the samples positioned next to each other on the wafer (Figure S5). Therefore, a nominal thickness gradient similar to that of the backside-coated samples used for ICR measurements can be assumed. Since the coating thickness is in the two-digit nanometer range, attenuation effects are assumed to play only a minor role, and the measured XRF Pt intensities are therefore considered proportional to the thickness of the sputtered platinum layer.

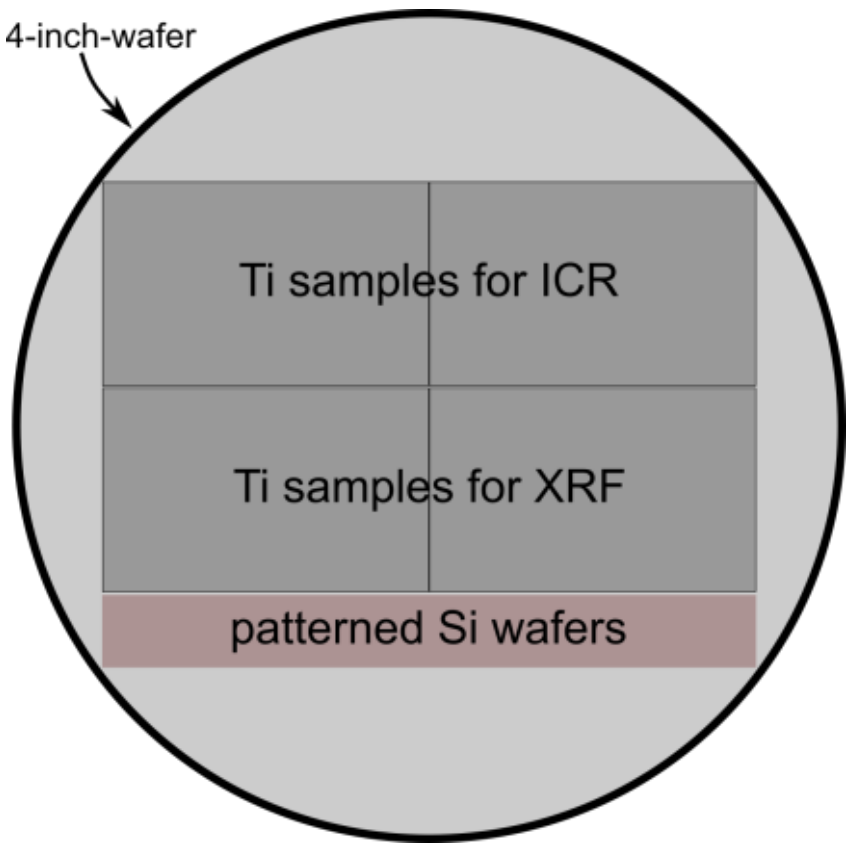

Figure S5. Schematic drawing of placing of the Ti samples for ICR and XRF testing (for each sample set two 40 x 25 mm Ti samples were placed next to each other) and the photolithographically patterned Si wafer on the wafer during deposition of the thin Pt-wedge.

2 Calibration measurement

Figure S6 displays the calibration measurement for various assemblies. The calibration value is highly reproducible per assembly. We have found it beneficial to use a break-in procedure by approaching the Cu-rod assembly with a higher compaction force than during measurement to accelerate stabilization of the calibration value, afterwards the calibration value stabilizes more quickly, which usually occurs within the first 50 measurements. Repeated testing of the calibration value resulted in similar values throughout extensive use of one GDL for sample measurements.

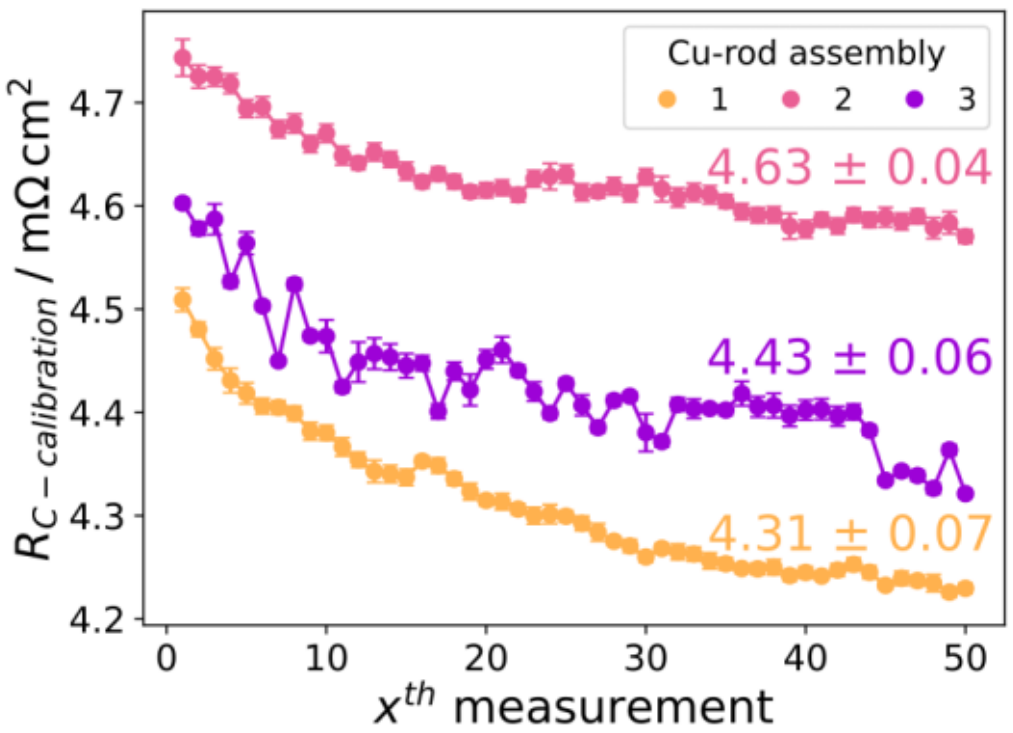

Figure S6. Calibration measurement for three different assemblies. 50x measurements on a single spot, with their average and standard deviation indicated in the plot.

The lifetime of the small GDL has been found to be up to 1,000 measurements and might even endure longer. However, it may occasionally fail after far fewer measurements; because the material is brittle, mechanical breakage can occur, necessitating replacement.

3 Pristine Materials

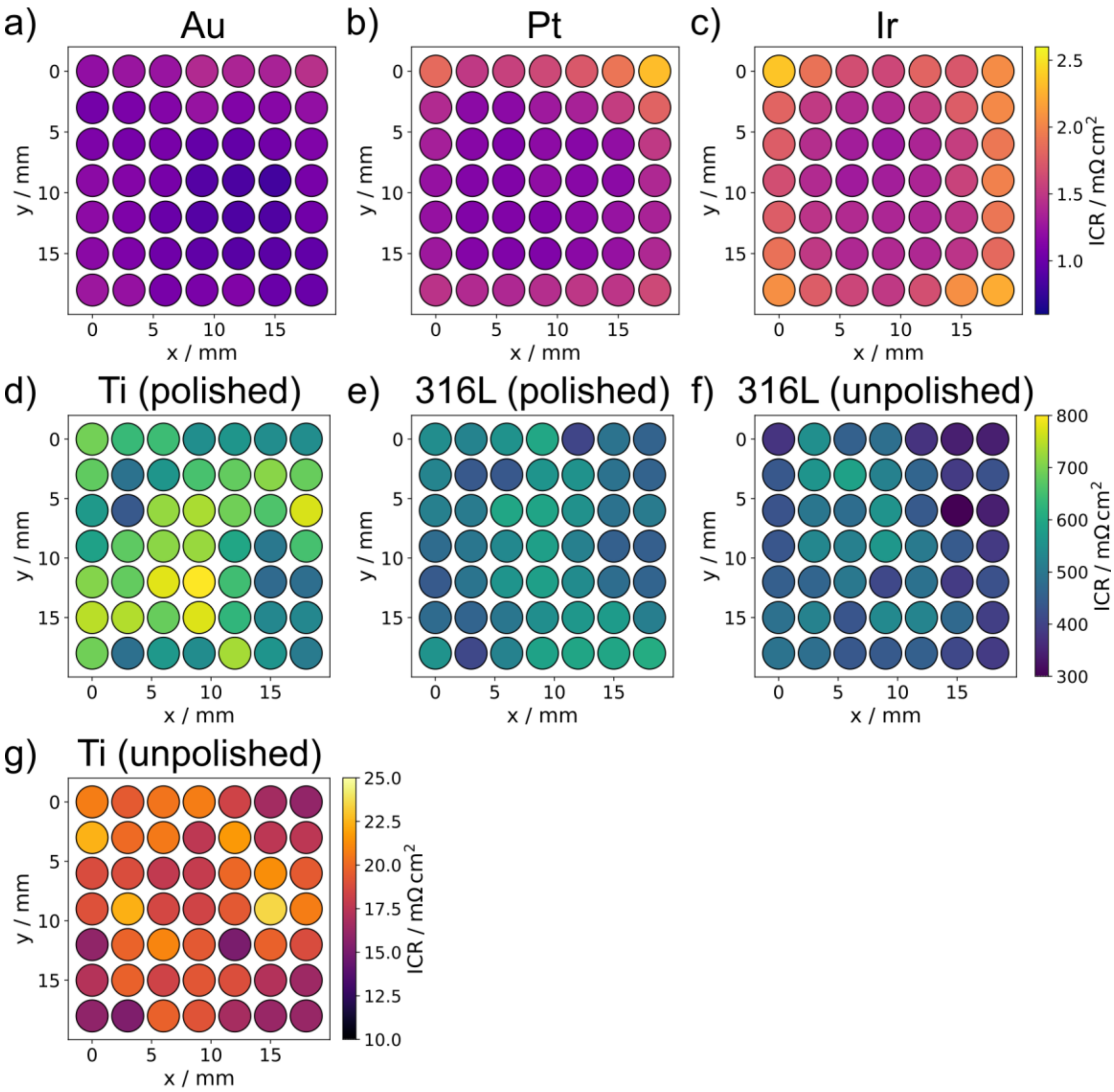

Figure S7. ICR measurement scanning across 49 spots (7x7 pattern with 3 mm x 3 mm distance, meander starting at (0|0)) per sample: a) Au, b) Pt, c) Ir, d) polished Ti, e) polished 316L, f) unpolished 316L, and g) unpolished Ti. Average with standard deviation per measurement is plotted in Figure 1 of the manuscript.

The ICR values of titanium with different surface roughnesses are shown in Figure S8. The lower the surface roughness of titanium, the higher the average ICR across the sample surface (Figure S4d–e). This trend has already been described beforehand in the literature for stainless steel.[16-17] It is likely caused by a higher contact area between the fiber of the GDL and the sample if the surface is rougher. The surface roughness of unpolished Ti is significantly higher than the surface roughness of polished Ti as well as polished and unpolished 316L (Figure S9), explaining the measured low ICR value of unpolished Ti (Figure 1 in manuscript). Therefore, to obtain a low ICR value, not only is the material choice important, but if a non-ideal material is chosen, its roughness can either beneficially or negatively influence the ICR value.

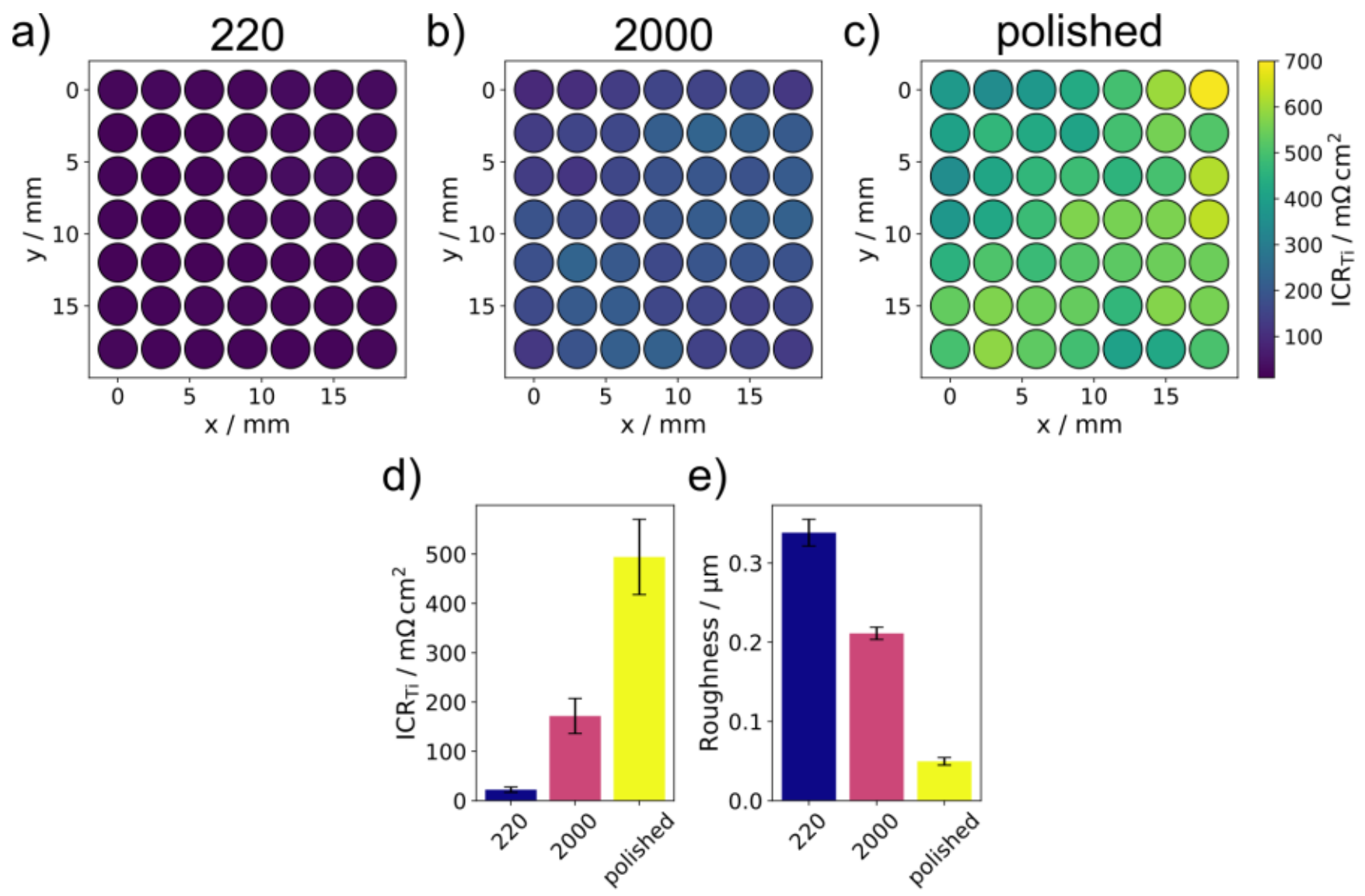

Figure S8. Influence of surface roughness on the ICR of Ti (t = 1.5 mm). Scan over 49 spots (7x7 pattern with 3 mm x3 mm distance, meander starting at (0|0)) per sample: a) Grinded with SiC-Paper #220, b) Grinded with SiC-Paper #2000, c) Polished with ¼ diamond suspension. d) Average ICR of measurements displayed in a – c. e) Surface area roughness of the samples.

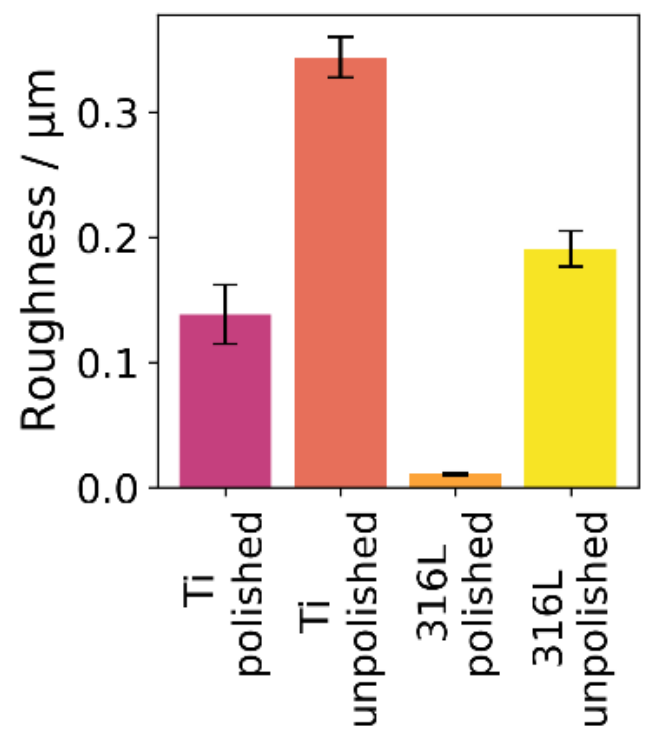

Figure S9. Area surface roughness of Ti and 316L (Evek) in its pristine and polished state determined by laser profilometry measurements. Average and standard deviation of three measurements on independent locations per sample.

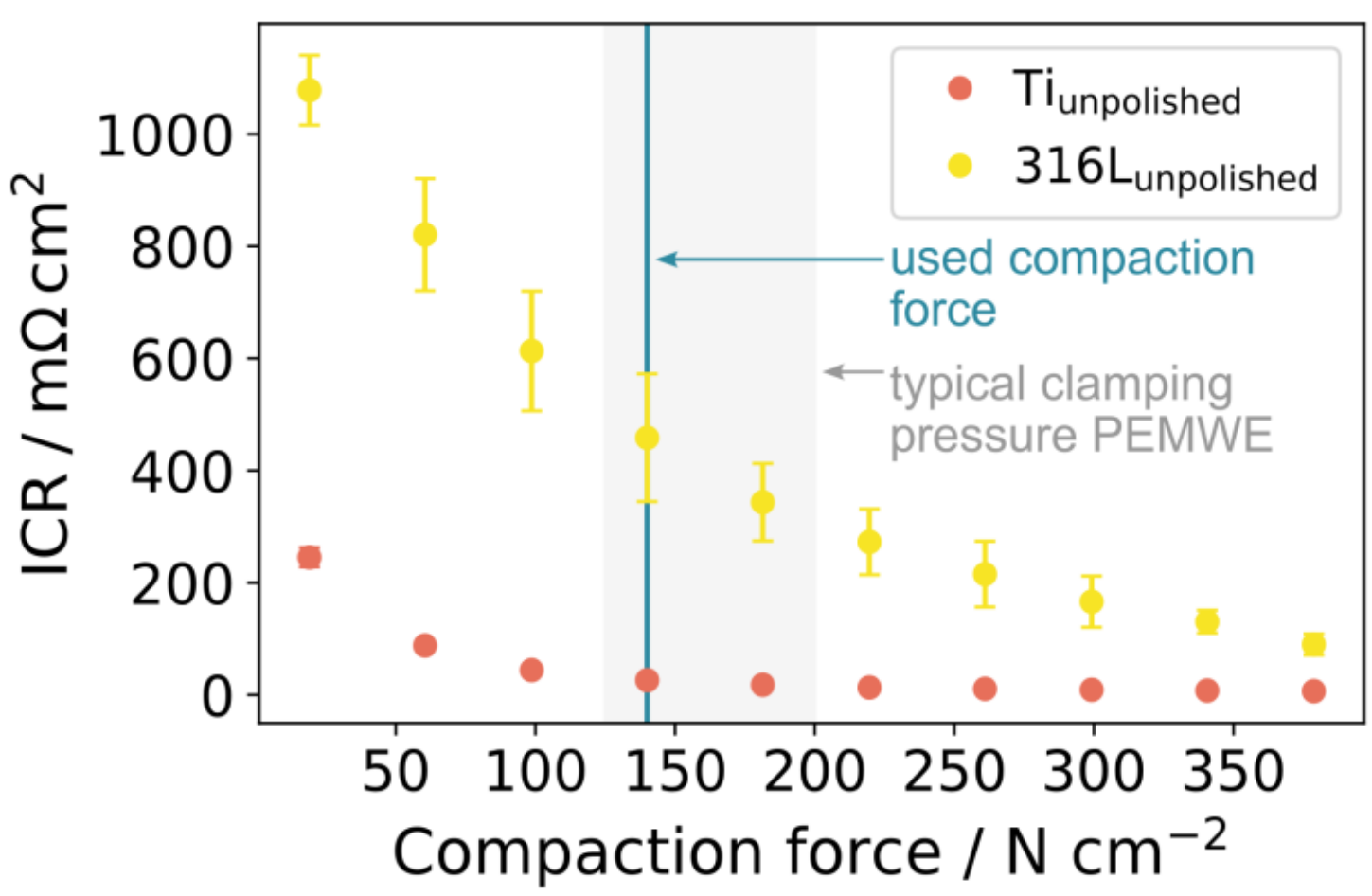

Figure S10. Compaction force dependence of the ICR of unpolished Ti and 316L. Average of three measurements with standard deviation per compression force p at three independent locations per sample. The gray area corresponds to typical compressions of PEM electrolyzers.[2] The blue line indicates the typically used ~140 N cm^{-2} in this study, which is the specified compaction force for ICR testing of BPP for fuel cell by the DOE.[18]

Figure S11 displays 50x repetitive measurement on a single sample spot. While noble metals (Figure S7a) show values that quickly stabilize after an initial increase (Pt) or decrease (Au and Ir), the behavior is more complex for Ti and 316L (Figure S11b). For both polished and unpolished Ti, the ICR value increases. For unpolished 316L, an initial increase is observed; however, a temporary decrease occurs after approximately 30 measurements. For polished 316L, an ICR increase within the first 6 measurements is observed, after which the value stabilizes. This results in similar ICR values for noble materials, whereas those of 316L and Ti are enhanced compared to scanning across the sample (Table S1 and Table S2).

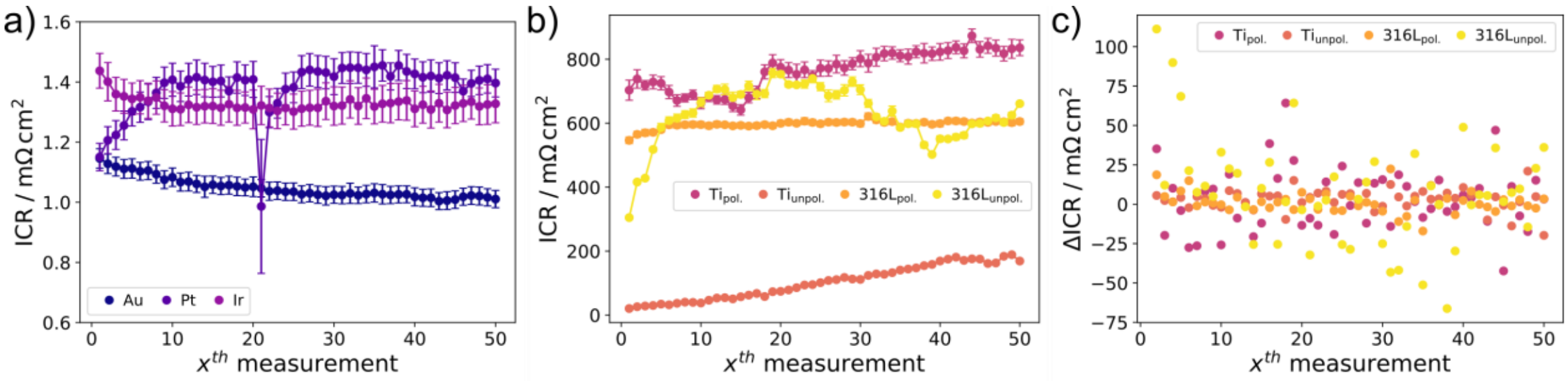

Figure S11. Repetitive ICR measurements on one single spot on pristine materials. a) Au, Pt, and Ir. b) Ti and 316L. c) Difference of ICR value from measurement to measurement of Ti and 316L displayed in c).

Table S1. ICR values of Au, Pt, and Ir. Average with standard deviation of the ICR measured across 49 different spots (Figure S7a – c) as well as 50 repetitive measurements on one spot (Figure S11a).

	ICR / mΩ cm²		
	Au	Pt	Ir
50x one spot	1.05 ± 0.05	1.37 ± 0.09	1.32 ± 0.03
49 spots	1.09 ± 0.15	1.35 ± 0.26	1.63 ± 0.27

Table S2. ICR values of Ti and 316L. Average with standard deviation of the ICR measured across 49 different spots (Figure S7d – g) as well as 50 repetitive measurements on one spot (Figure S11b).

	ICR / mΩ cm²			
	Ti$_{polished}$	Ti$_{unpolished}$	316L$_{polished}$	316L$_{unpolished}$
50x one spot	765.1 ± 62.3	102.2 ± 53.6	596.7 ± 12.3	629.7 ± 91.1
49 spots	631.2 ± 95.6	18.8 ± 2.0	516.5 ± 54.9	454.6 ± 65.8

Additionally, we have tested the reproducibility between assemblies for the measurement of an unpolished Ti sample. Even though the average ICR values for the both independent scans is similar (Table S3), the local ICR between runs changes (Figure S12).

Table S3. Average ICR value with standard deviation of ICR value scanning across 84 spots (12x7 pattern with 3 mm x 3 mm distance) across unpolished Ti (Evek). Between the two scans, the setup was newly assembled. The difference between the single measurement spots is displayed in Figure S12.

	ICR / mΩ cm²	
	Ti$_{unpolished}$ – Sample 1	Ti$_{unpolished}$ – Sample 2
Scan 1	23.2 ± 3.9	22.6 ± 2.9
Scan 2	24.3 ± 1.5	26.7 ± 4.1

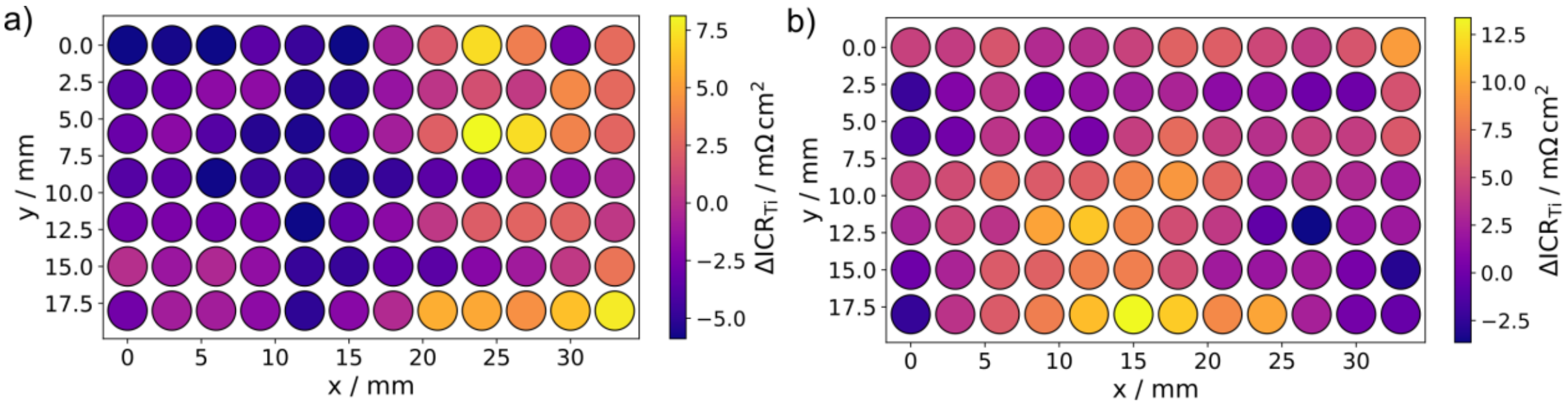

Figure S12. Local ICR difference between two scans across 84 spots (12x7 pattern with 3 mm x 3 mm distance, meander starting at (0|0)) per sample) with disassembly between the scans for two different (a and b) Ti samples (Evek, unpolished, 25x40 mm).

4 Corroded Materials

SFC-ICP-MS measurement over 600 s of unpolished Ti in 10 mM H_2SO_4 at 9 different potentials are shown in Figure S13. For all measurements, the current density rapidly drops and stabilizes below 0.033 ± 0.013 mA cm^{-2}. The higher the applied potential, the higher is the initial current density as well as the current density average over the last 20 s of the measurement (Figure S14). Upon potential application, the dissolution rate of Ti increases to a maximum within the first 100 s, after which the dissolution rate decreases throughout the rest of the measurement time (Figure S13).

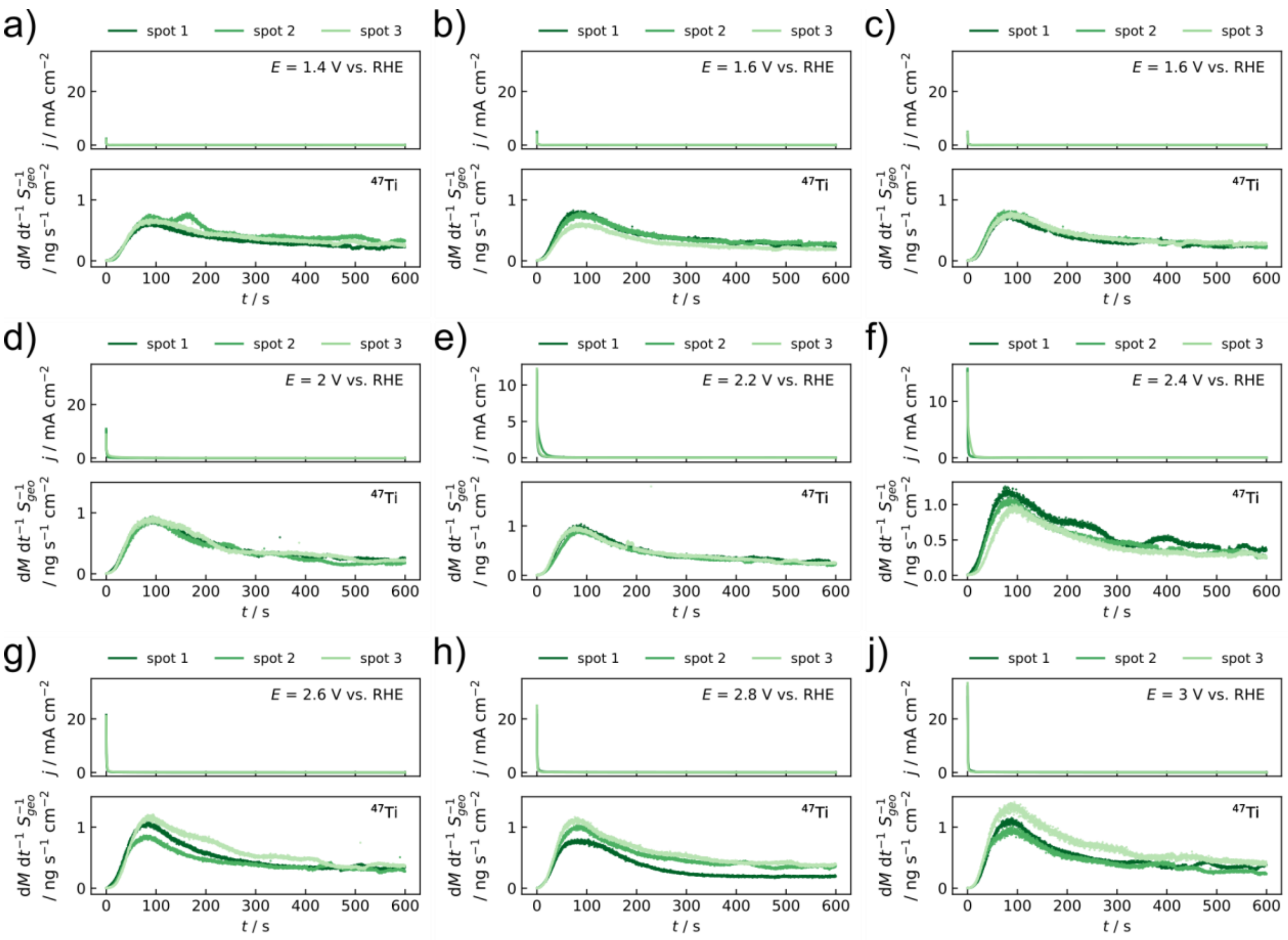

Figure S13. SFC-ICP-MS measurements of Ti (unpolished). Potentiostatic holds over 600 s in 10 mM H_2SO_4 at T_{stage} = 60 °C. Applied potential indicted in plots (a – j). From top to bottom: Current density and dissolution rate of Ti. Per potential, three independent, pristine spots were measured (dark to light).

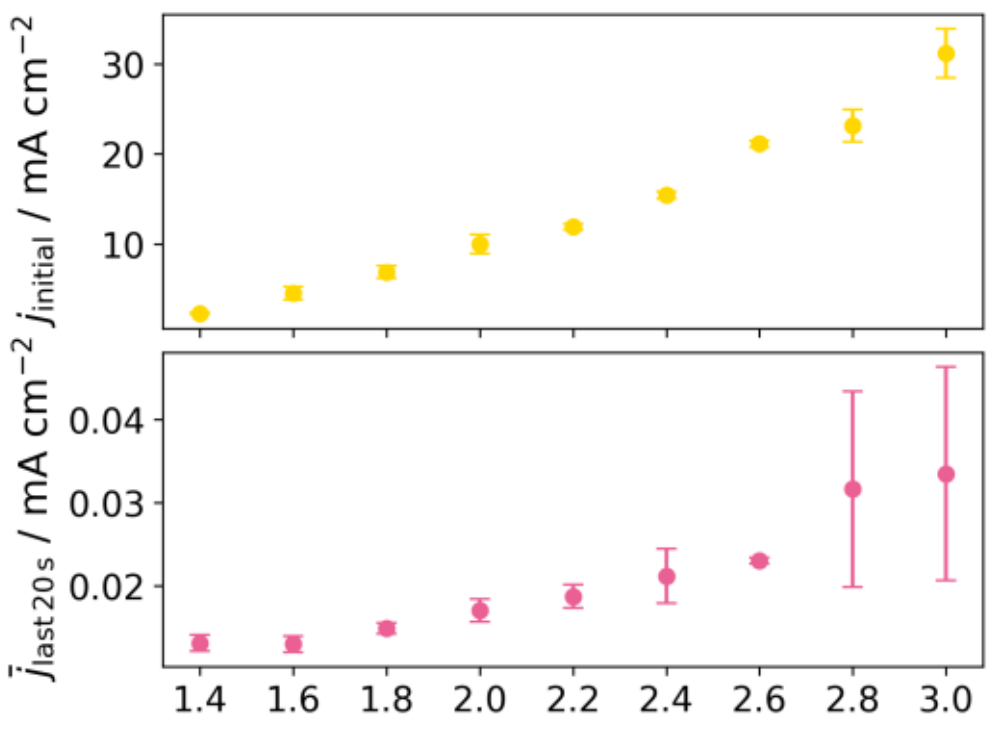

Figure S14. Initial current density and average over the last 20 s during SFC-ICP-MS measurements of Ti displayed in Figure S13 (average and standard deviation of three measurements on independent sample locations).

From the SFC-ICP-MS data, we have calculated the dissolution efficiency (DE) using the method presented by Göhl *et al*.[19] and as already done in our previous study.[15]

In short, the DE is defined as the ratio of faradaic current Q_{Diss} to oxidize the sample with subsequent dissolution to the total transferred charge during the SFC-ICP-MS measurement Q_{EC} (Equation (16)).

$$DE = 100\% \cdot \frac{Q_{Diss}}{Q_{EC}} = 100\% \cdot \frac{\frac{m_{Diss}}{M} \cdot zF}{\int I\,dt} \quad (16)$$

With m_{Diss} as the dissolved mass during the SFC-ICP-MS measurement, z as transferred electrons, F as Faraday constant, M as molar mass, I as the recorded current, and t as time.

The assumed oxidation reaction for Ti is its oxidation to TiO_2 (Equation (17)).[20]

$$\mathrm{Ti} + 2\,\mathrm{H_2O} \rightarrow \mathrm{TiO_2} + 4\,\mathrm{H^+} + 4\,\mathrm{e^-} \quad (17)$$

The ICR of Ti after applying various potentials is shown for each measurement spot in Figure S15a. As different Ti samples have been used for SFC-ICP-MS experiments, multiple pristine values are shown, all being within the same range. For each sample, the average ICR value across the entire sample was taken as the pristine value. To better visualize the ICR change caused by corrosion, the change compared to the pristine value is shown in Figure S15b.

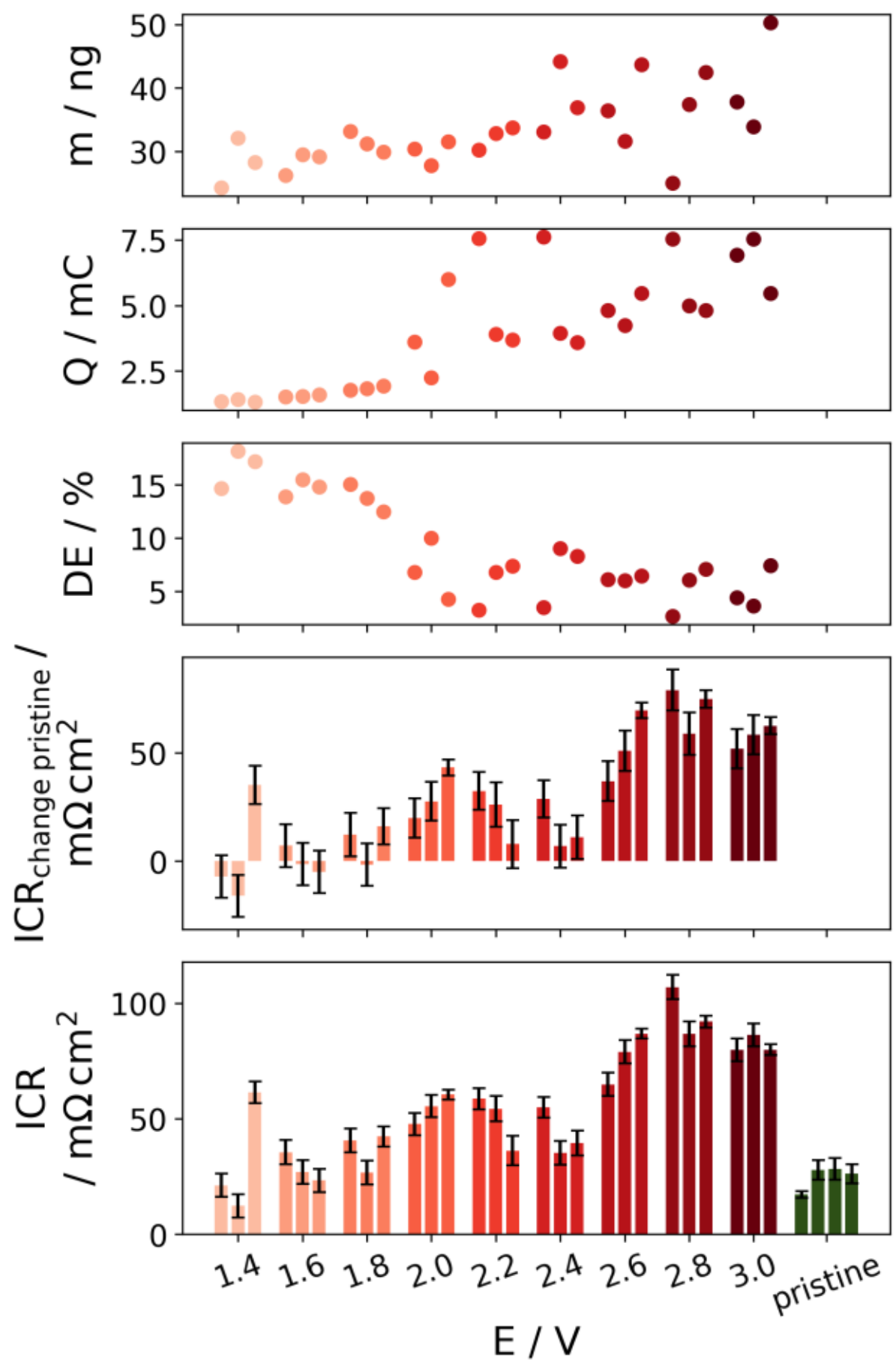

Figure S15. Correlative ICR measurement of Ti after oxidation at various potentials. Per potential three measurements on independent sample locations were conducted. The average and standard deviation of the single measurements is displayed in Figure 2b and c in the manuscript. From top to bottom: Analysis of SFC-ICP-MS measurements displayed in Figure S8: dissolved mass m_{Ti} and transferred charge Q during the measurement as well as the dissolution efficiency, ICR measurements showing the ICR change compared to its pristine value, and the absolute ICR depending on the applied potential.) Absolute ICR value, and b) relative change to pristine value. Per applied potential, three independent spots on the sample were measured.

5 Pt-wedges on Titanium

We have calculated the platinum group metal (PGM) loading of Pt-coated BPPs depending on the thickness of the Pt-coating (Equation (18), Figure S16). The horizontal lines correspond to the total PGM content targets for PEMWE specified by the U.S. Department of Energy (DOE).[21] It is important to note that these targets refer to the overall PGM content of the stack, i.e., the sum of the catalyst layers on both electrodes and any additional PGM coatings (such as those on the porous transport layer). The calculation shows that, to meet the DOE targets, a Pt coating on the BPP must be extremely thin.

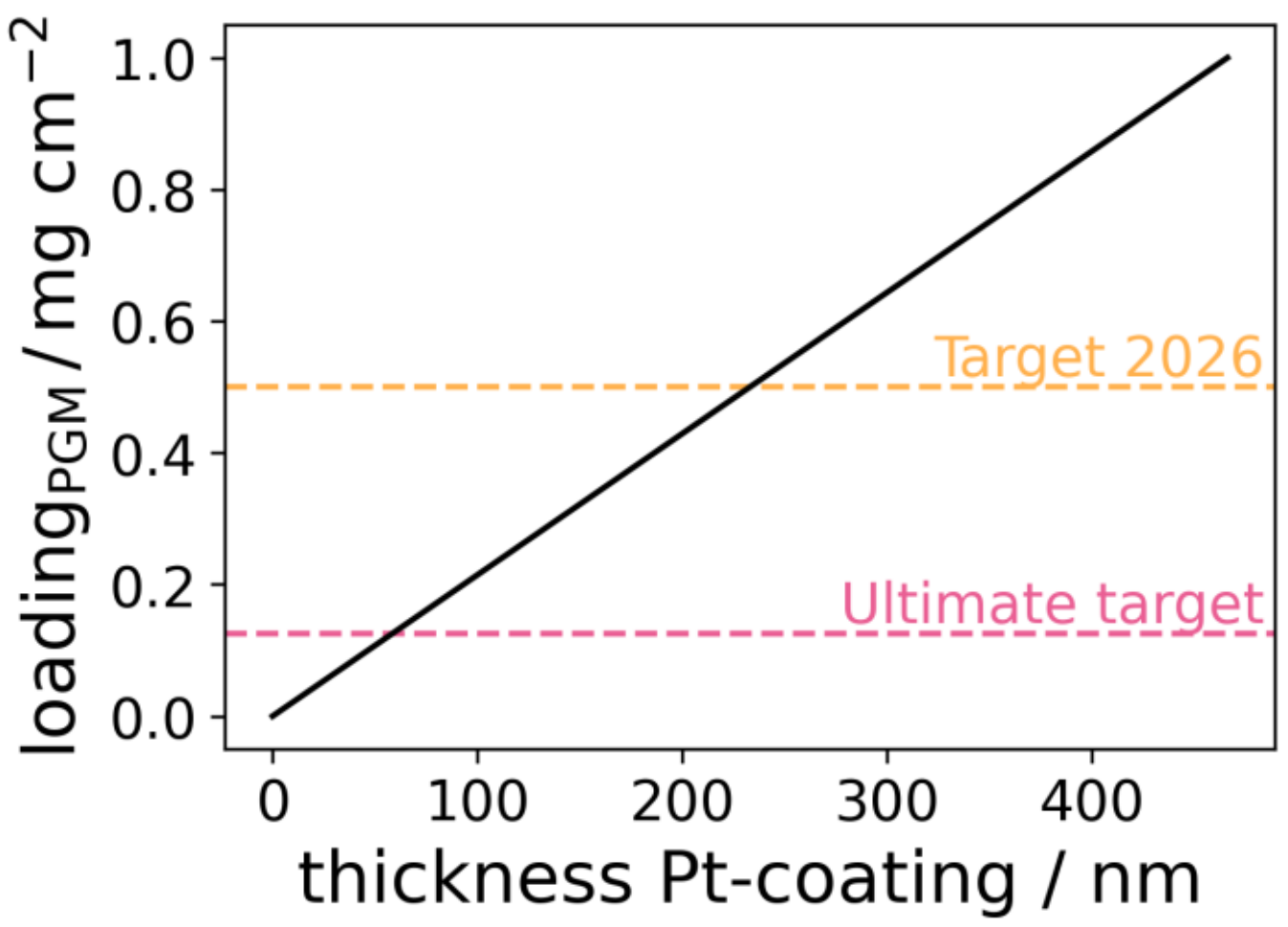

Figure S16. Dependence of PGM loading on Pt coating thickness. Dashed lines mark the DOE-specified PGM-content targets.[21]

$$loading_{PGM} = thickness\ Pt\ coating\ \cdot \rho_{Pt} \qquad (18)$$

With ρ_{Pt} as density of Pt (21.45 mg cm^{-2}).

Figure S17 shows an overview of the Pt-coated Ti-sheets (unpolished Ti). To investigate the influence of a Pt coating on the ICR, we have prepared two sample sets with a wedge-type Pt thickness gradient an 80 mm length (~0 – 33 nm_{Pt} and ~10 – 83 nm_{Pt}). As the sample size of the ICR setup is limited to samples of a maximum dimension of 50 mm, two 40 mm Ti sheets have been placed side-by-side during PVD deposition. Prior to Pt, a thin Ti adhesion layer was deposited to improve Pt Ti bonding. As we have suspected that the differences and overall ICR values of Pt-coated Ti would exhibit only a few mΩ cm² of ICR (i.e., a low-range signal), the backsides of the samples were also coated with a uniform Pt layer (16 ± 2.8 nm_{Pt}) to obtain a low backside ICR with low standard deviation. As during ICR testing, the ICR of both sides of the samples is measured (refer to Section 1.1, Supporting Information, for more details), we have additionally prepared a reference sample with a 16 ± 3.5 nm Pt coating on both sides. This reference shows a homogeneous ICR of 0.6 ± 0.1 mΩ cm² across the surface (Figure S18). The measured average value was used to correct the backside contribution of the wedge-type samples during ICR testing (Equation (9)).

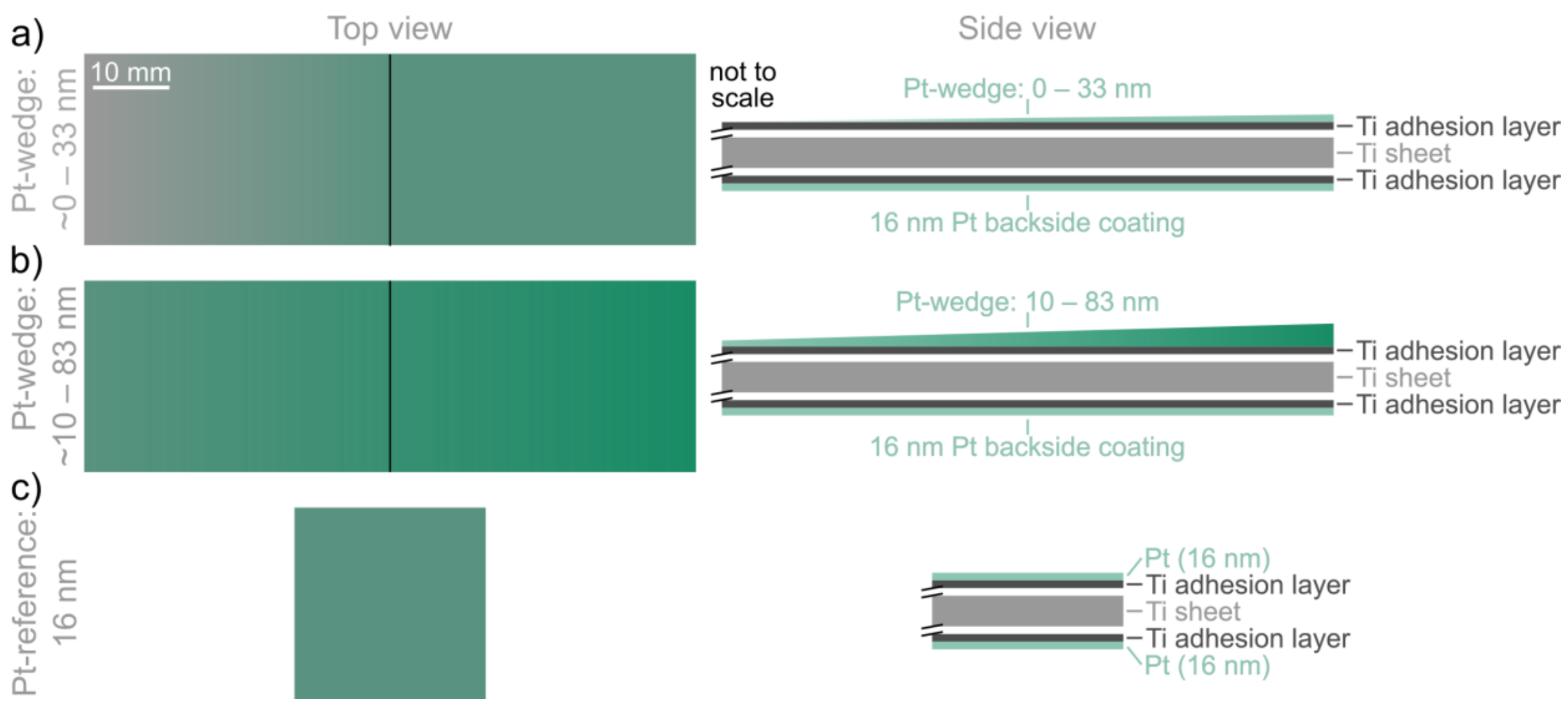

Figure S17. Overview of Pt-coated Ti samples (top and side view). a) Thin wedge with a Pt thickness gradient (0 – 33 nm_{Pt}) on top side across two 40 mm-long Ti sheets, b) Thick wedge with a Pt thickness gradient (10 – 83 nm_{Pt}) on top side across two 40 mm-long Ti sheets, and c) Reference sample with a uniform 16 nm Pt reference layer on top side. The backside of all samples is coated with a uniform 16 nm Pt layer. Below all Pt coatings, a 20 nm Ti adhesion layer has been deposited on the Ti sheets.

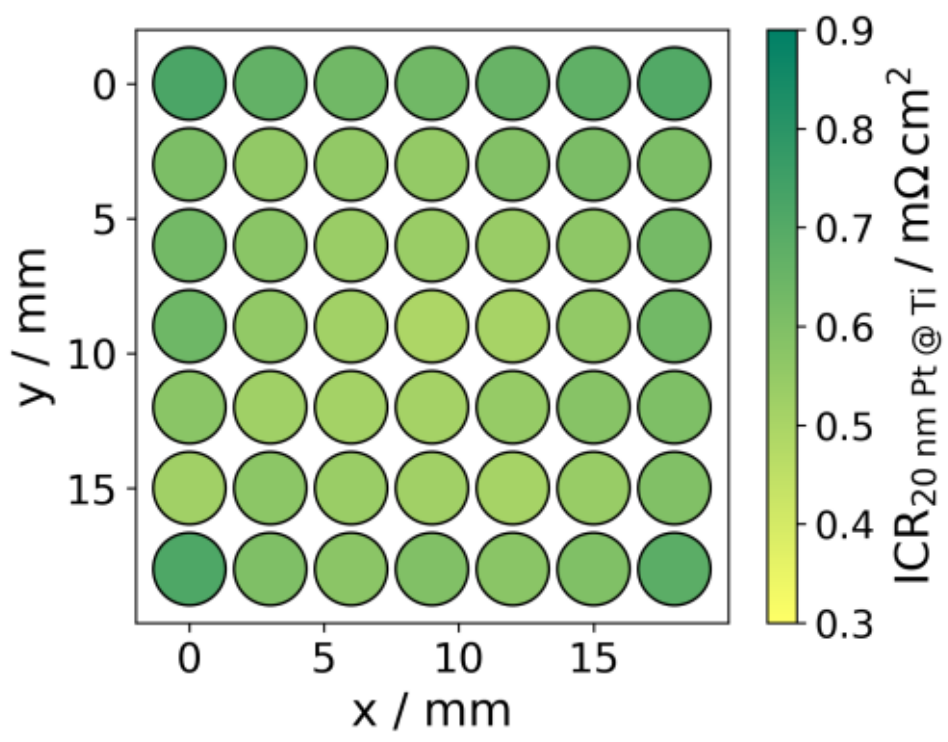

Figure S18. ICR measurement scanning across 49 spots (7x7 pattern with 3 mm x 3 mm distance, meander starting at (0|0)) over Pt-coated (thickness 16 ± 2.8 nm_{Pt}) Ti. Both sides of the sample have been coated. Average ICR: 0.6 ± 0.1 mΩ cm^2.

The Pt-thickness gradients across the samples are quantified by profilometry (see the Experimental Section for details). Especially due to the low Pt thickness on the thin wedge type sample combined with the relatively high surface roughness of the Ti substrates, the photolithographically patterned Si witness wafer provided the most reliable method for determining the Pt coating thickness. Cross sectional SEM does not provide sufficient resolution for accurate thickness determination while the substrate roughness prevents reliable measurements by reflectance spectroscopy or atomic force microscopy.

Because a Ti adhesion layer was deposited beneath the Pt, the raw profilometry values must be corrected for the Ti layer thickness. Figure S19a displays the thickness distribution of the Ti-adhesion layer. From the Ti thickness measured at the wafer centre, a sputter rate of 0.054 nm_{Ti} s^{-1} is obtained. To reach a nominal 20 nm Ti thickness, the sputter time was reduced by a factor of 0.13 for the deposition of the Ti-adhesion layer. We chose to perform profilometry measurements to determine the sputter rate of Ti and the thickness distribution across the wafer on a thicker Ti layer to improve the accuracy of the profilometry measurements. All critical deposition parameters are computer controlled so that after ignition and pre-sputtering the deposition rate is constant. Consequently, the Ti thickness scales linearly with the sputtering time, and reducing the deposition time by a factor of 0.13 reduces the resulting Ti thickness by the same factor. The 20 nm Ti thickness distribution was therefore calculated by multiplying the thickness profile of the thicker Ti layer by a scaling factor of 0.13 (Figure S19b). The purple rectangles indicate the position of the Si substrate during Ti/Pt deposition relative to the wafer centre. Although only a single Si piece was used, Ti thicknesses were averaged at equidistant points from the wafer centre to minimize error, as the profilometry confirmed a radially symmetric thickness profile. The resulting Ti-adhesion-layer thickness along the wafer is plotted in Figure S19c. These values were subsequently used to correct the total thickness measured after Pt deposition (Equations (19) and (20)).

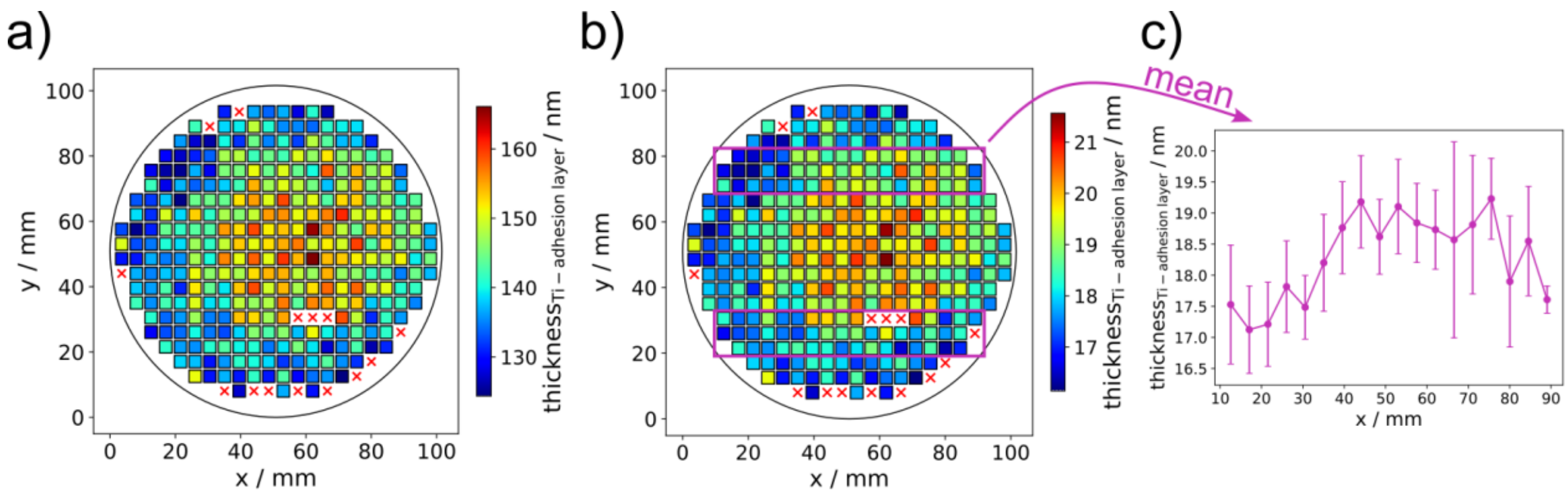

Figure S19. Profilometry measurements to obtain the thickness distribution of the Ti adhesion layer across the wafer. a) Thickness distribution of a 155 nm Ti adhesion layer. b) Thickness distribution of a 20 nm Ti adhesion layer based on the thickness distribution shown in a). The same factor used to adjust the thickness during sputtering was used to calculate the thickness distribution. Purple squares indicate the location of the Si wafer for thickness determination. c) Average standard deviation of the thickness of Ti adhesion layer across the wafer (based on marked area (purple) in b)).

$$thickness_{Pt} = thickness_{profilometry} - thickness_{Ti-adhesion\ layer} \tag{19}$$

$$\sigma(thickness_{Pt}) = \sqrt{\sigma\left(thickness_{profilometry}\right)^2 + \sigma\left(thickness_{Ti-adhesion\ layer}\right)^2} \tag{20}$$

Figure S20 presents the profilometry measurements for the Pt-wedge samples. The Pt thickness at each point was obtained by subtracting the local Ti-adhesion layer thickness (Figure S19b) from the measured thickness (Equations (19) and (20)). A linear regression was then applied, as the deposition parameters were chosen to produce a linear gradient (Section 1.6, Supporting Information). This fit is indicated in Figure S20 (bottom). The achieved Pt gradients are: 0 – 33 ± 1.8 nm$_{Pt}$ and 10 ± 1.8 – 83 ± 1.8 nm$_{Pt}$, respectively.
For the thin wedge, the shutter movement left a small region (~x ≤ 3 mm) completely uncoated, giving a nominal 0 nm Pt thickness. At the thick end, the Pt thickness is 33 ± 1.8 nm$_{Pt}$.

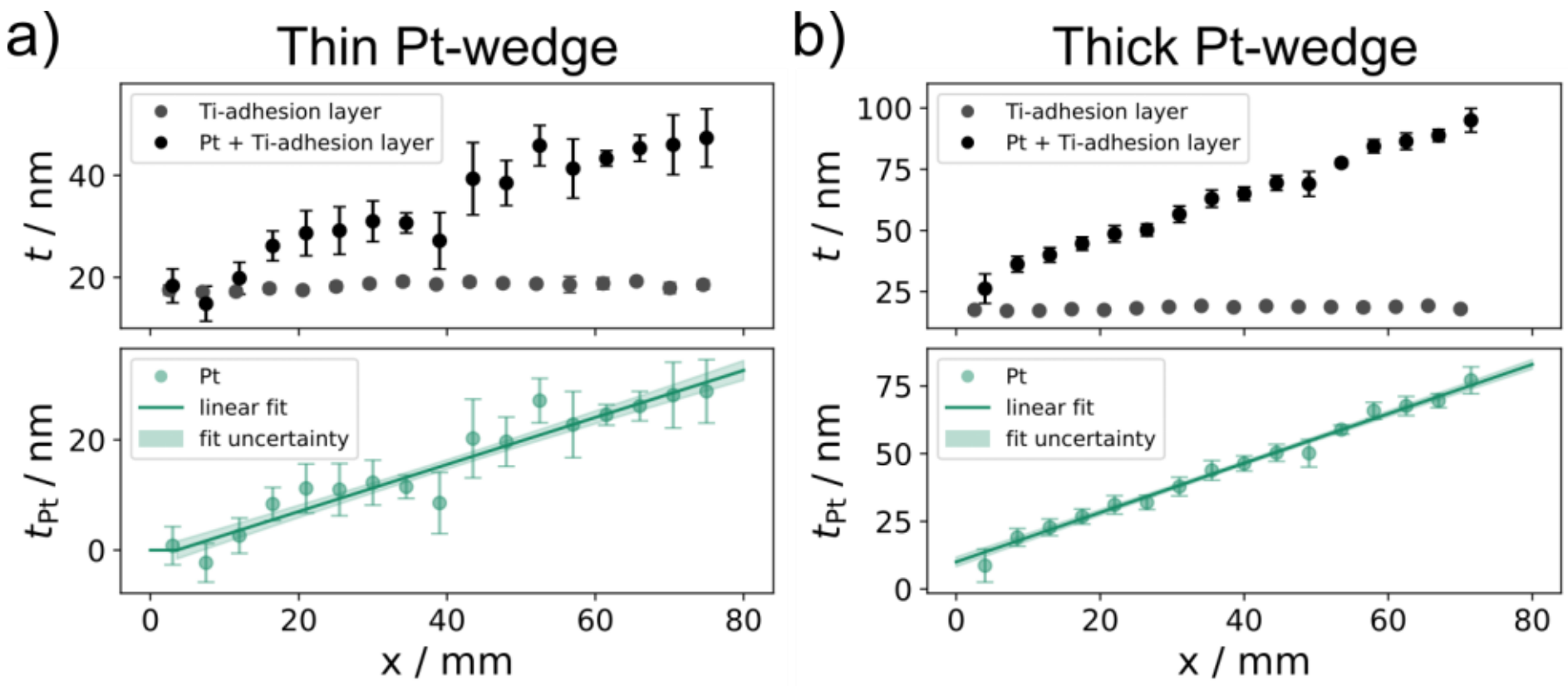

Figure S20. Thickness distribution across the Pt-coated Ti sample. a) Thin wedge (0 – 33 ± 1.8 nm$_{Pt}$). b) Thick wedge (10 ± 1.8 – 83 ± 1.8 nm$_{Pt}$). Top: thickness of 20 nm Ti adhesion layer and measured thickness of deposited Ti adhesion layer with Pt by profilometry. Bottom: calculated Pt thickness across Ti with its linear fit (thin wedge: t_{Pt} = (0.43 ± 0.04 nm$_{Pt}$) x – (1.52 ± 1.72 nm$_{Pt}$); thick wedge: t_{Pt} = (0.91 ± 0.04 nm$_{Pt}$) x + (10.04 ± 1.78 nm$_{Pt}$)).

The ICR profile across the thin wedge-type Pt-coated Ti sample was analyzed using a piecewise linear regression approach. Three overlapping data regions (high ICR between x = 0 – 7.5 mm, transition region between x = 7.5 – 12.5 mm and low ICR region x ≥ 12.5 mm) were independently fitted by a weighted linear regression (Figure S21). The transition points were determined from the intersections of adjacent fitted regression lines. By using the linear fit of the Pt-thickness across the sample (Figure S20), the second transition point, beyond which no further significant improvement in ICR was observed, corresponds to a Pt thickness of 3.8 ± 1.4 nm$_{Pt}$.

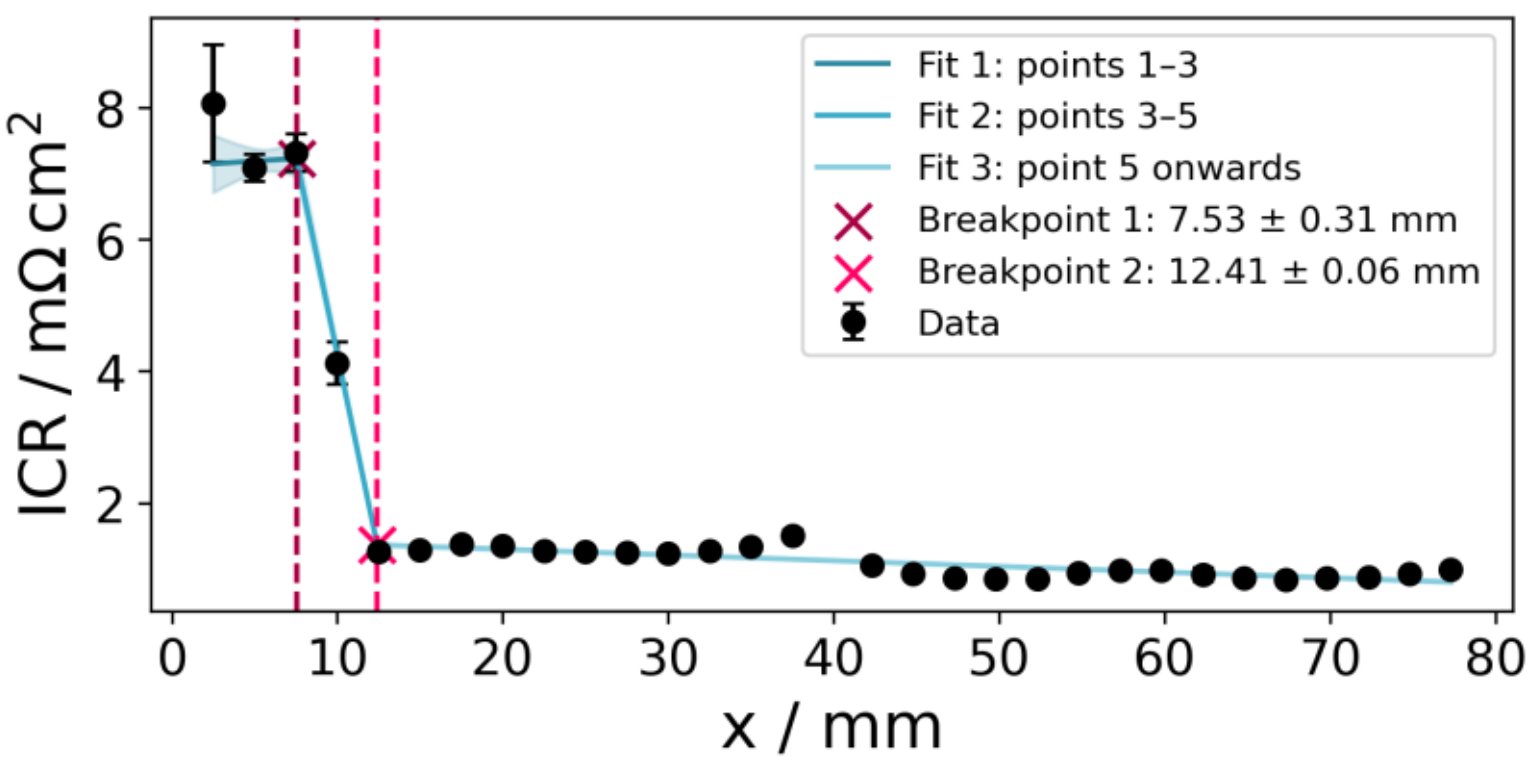

Figure S21. Segmented linear regression of the ICR measurements (sampling diameter: 2 mm) across Pt-coated Ti. Topside: ~0 – 33 ± 1.8 nm$_{Pt}$, backside: 16 ± 2.8 nm Pt coating. The average with standard deviation of 5 measurements at a location with identical Pt-thickness of is plotted (individual measurements displayed in Figure 3).

To further validate the thin wedge-type Pt-thickness gradient on Ti, we have performed XRF measurements (Figure S22, green). At positions x < 10 mm, Pt was detected only sporadically, indicating the presence of isolated Pt islands on the Ti surface. At measurements positions x > 10 mm, the Pt signal increases steadily.

The XRF signal of the reference sample (Figure S22, gray) matches that of the wedge at x ≈ 40mm. Using the linear fit of the thin wedge (Figure S20a), yields a Pt thickness of 15.5 ± 0.9 nm_{Pt} at this position. The thickness of the reference sample was independently determined to be 16 ± 2.8 nm_{Pt} by profilometry, indicating good agreement with the linear fit of the thin wedge.

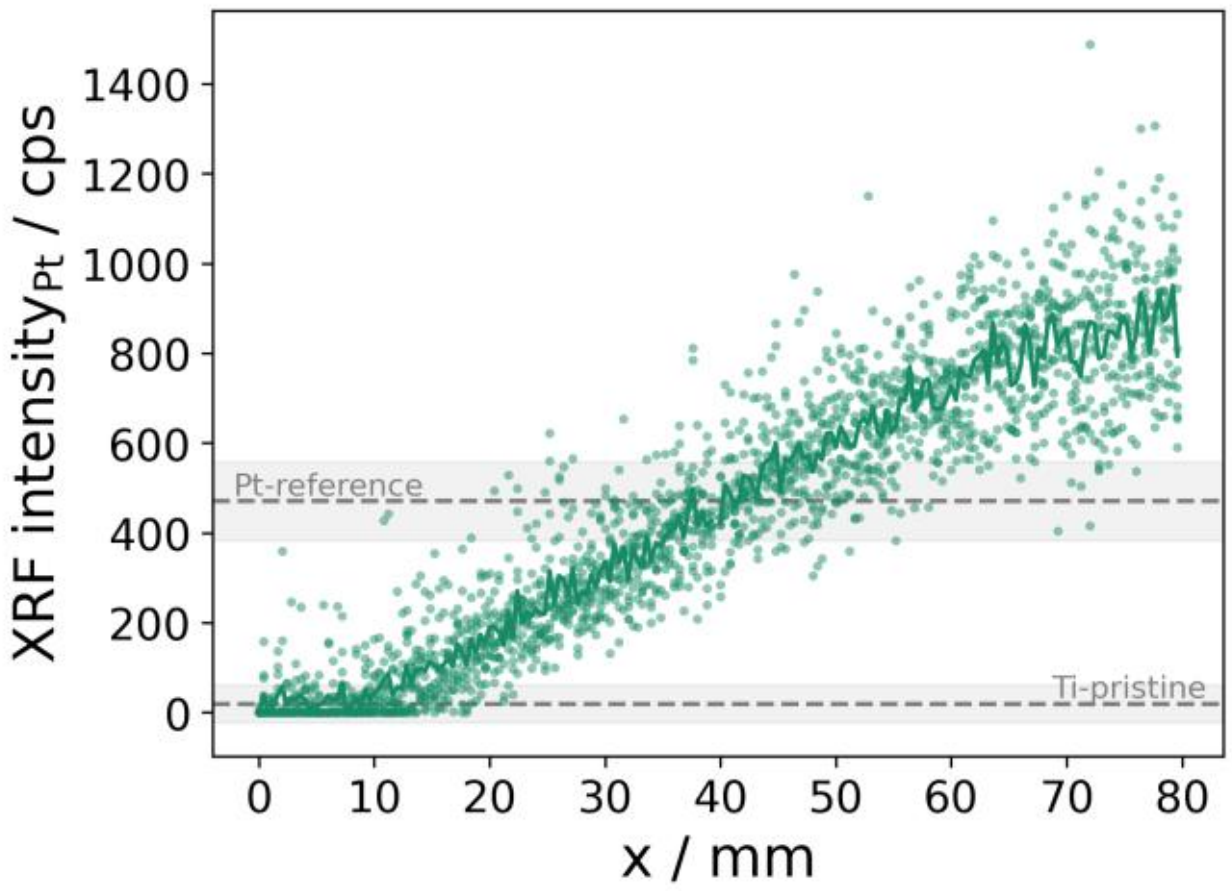

Figure S22. XRF measurements. Green: Pt-coated Ti with Pt wedge-type thickness gradient (thin wedge: 0 – 33 ± 1.8 nm_{Pt}) – single measurements (10 per x-location) and their average (dots and line, respectively). Gray: Homogenously Pt-coated Ti (16 ± 2.8 nm_{Pt}) and pristine titanium – average with standard deviation.

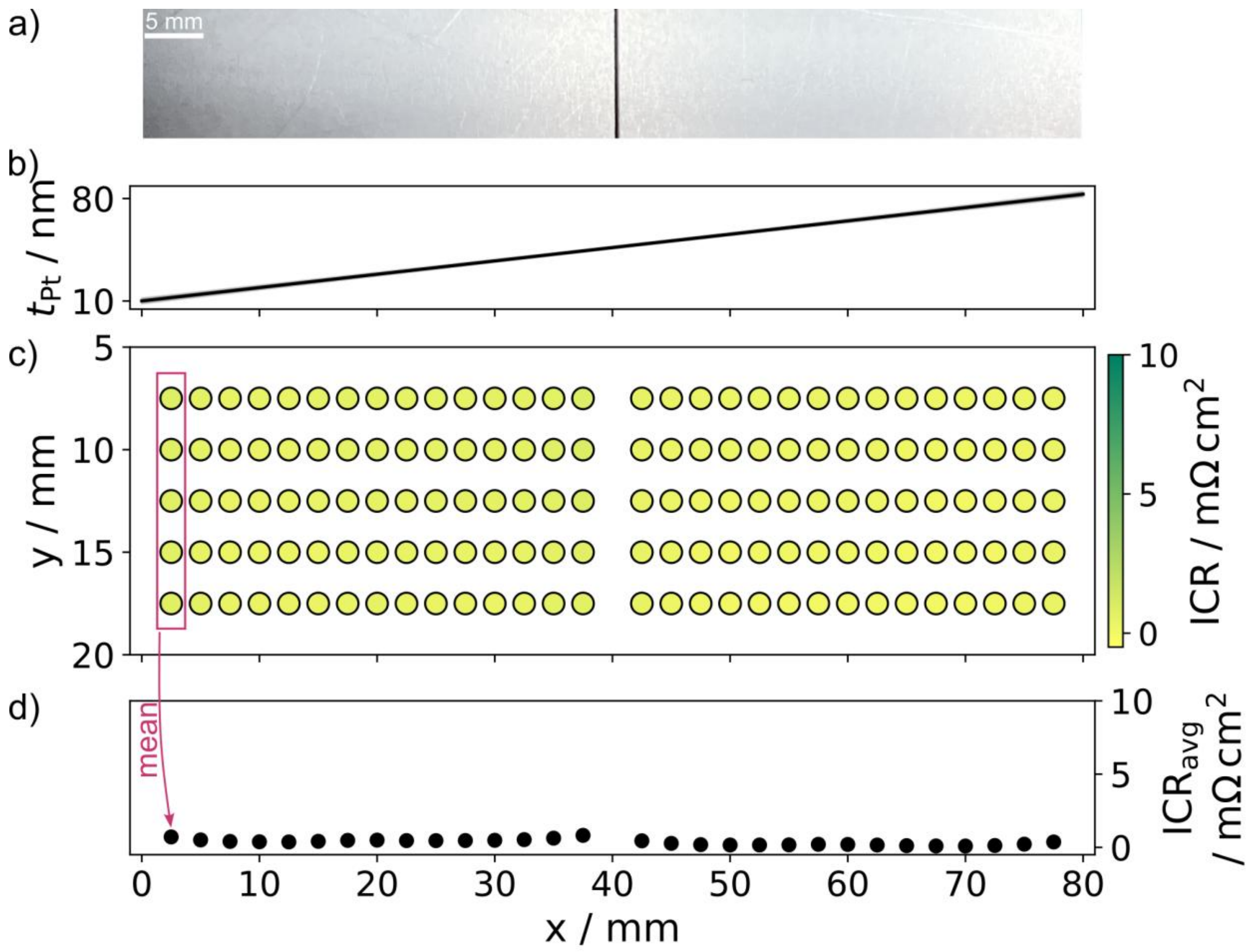

Figure S23. ICR measurements (sampling diameter: 2 mm) across Pt coated Ti. Topside: 10 ± 1.8 – 83 ± 1.8 nm_{Pt}, backside: 16 ± 2.8 nm Pt coating. a) Micrographs of the Pt wedge across the sample. b) Linear fit with uncertainty of Pt thickness gradient across sample (refer to Section 5, Supporting Information for further details). c) ICR measurements across the sample at different locations and d) average with standard deviation of 5 measurements at a location with identical Pt thickness of c).

The two possible current pathways during the ICR measurement are depicted in Figure S24. The current can either pass through the interface GDL-Ti or GDL-Pt-Ti (Figure S24a). Assuming a simple equivalent circuit and Kirchhoff's circuit laws, the effective ICR depends on the interfacial contact resistance as well as on the surface coverage of Pt and Ti, respectively (Figure S24b, Equations (21) and (22)). Assuming an ICR of 7 and 1 mΩ cm^{-2} for Ti and Pt-coated Ti, respectively (see manuscript), the estimated effective ICR is plotted in Figure S24c. It becomes apparent that, already at a coverage of 20 $\%_{Pt}$ on the surface of Ti, the ICR is estimated to decrease to 3.2 mΩ cm^2 and at a coverage of 50 $\%_{Pt}$ to 1.8 mΩ cm^2. If the difference between the "worse" and "good" ICR were even higher, the effect would be even more pronounced. We acknowledge that this, of course, does not consider the GDL's porosity, which could affect the actual surface area. However, we believe this provides the reader with a better understanding of how quickly the material with the lower ICR dominates the effective ICR.

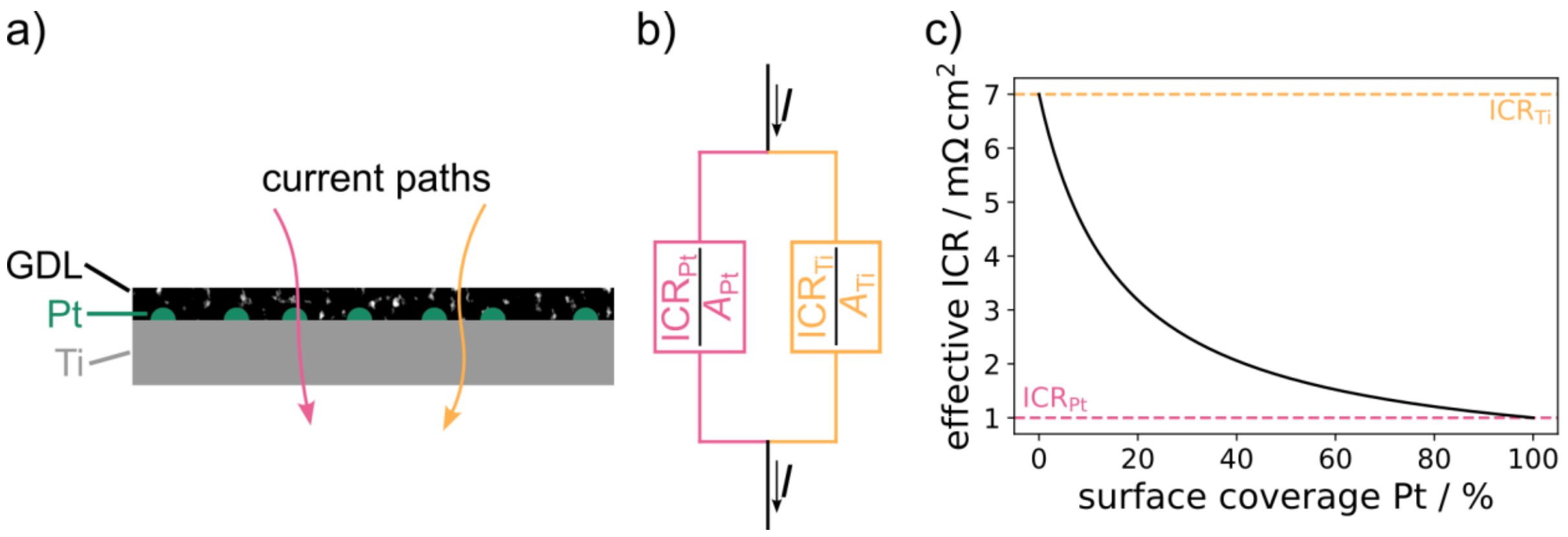

Figure S24. a) Sketch of current pathways through the interface for a non-fully covering Pt-coating on Ti, the current can pass through the interfaces GDL-Pt-Ti (pink) or Ti-GDL (yellow). b) Equivalent circuit of the interface assuming the ICR to be the dominating resistance per current path. c) Dependence of effective resistance on the Pt coverage on Ti.

$$\frac{1}{R_{eff}} = \frac{1}{R_{Ti}} + \frac{1}{R_{Pt}} = \frac{A_{Ti}}{ICR_{Ti}} + \frac{A_{Pt}}{ICR_{Pt}} = \frac{(1 - surface\ coverage_{Pt}) \cdot A}{ICR_{Ti}} + \frac{surface\ coverage_{Pt} \cdot A}{ICR_{Pt}} \tag{21}$$

$$ICR_{effective} = \frac{1}{\left(\frac{1 - surface\ coverage_{Pt}}{ICR_{Ti}} + \frac{surface\ coverage_{Pt}}{ICR_{Pt}}\right)} \tag{22}$$

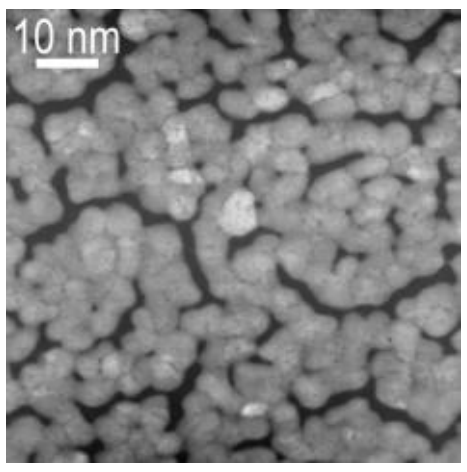

Figure S25. Top-view transmission electron microscopy (TEM) image of sputtered Pt (nominal thickness: 4.7 nm, determined by weighing.) on Si_3N_4 grids as reference. The Pt has a surface coverage of approximately 85%.

References

[1] W. Yoon, X. Huang, P. Fazzino, K. L. Reifsnider, M. A. Akkaoui, "Evaluation of coated metallic bipolar plates for polymer electrolyte membrane fuel cells" *J. Power Sources* **2008**, *179*, 265–273. 10.1016/j.jpowsour.2007.12.034.

[2] P. Lettenmeier, R. Wang, R. Abouatallah, B. Saruhan, O. Freitag, P. Gazdzicki, T. Morawietz, R. Hiesgen, A. S. Gago, K. A. Friedrich, "Low-Cost and Durable Bipolar Plates for Proton Exchange Membrane Electrolyzers" *Sci. Rep.* **2017**, *7*, 44035. 10.1038/srep44035.

[3] L. Messing, K. Pellumbi, L. Hoof, N. Imming, S. Wilbers, L. Kopietz, M. Joemann, A. Grevé, K. junge Puring, U. P. Apfel, "Carbon Bipolar Plates in PEM Water Electrolysis: Bust or Must?" *Adv. Energy Mater.* **2024**, *14*, 2402308. 10.1002/aenm.202402308.

[4] H. Ye, Z. Tu, S. Li, "Electrochemical performance of metal nitride coated titanium bipolar plate for proton exchange membrane water electrolyser" *J. Power Sources* **2024**, *595*, 234052. 10.1016/j.jpowsour.2024.234052.

[5] M. Forouzanmehr, K. Reza Kashyzadeh, A. Borjali, A. Ivanov, M. Jafarnode, T. H. Gan, B. Wang, M. Chizari, "Detection and Analysis of Corrosion and Contact Resistance Faults of TiN and CrN Coatings on 410 Stainless Steel as Bipolar Plates in PEM Fuel Cells" *Sensors* **2022**, *22*, 750. 10.3390/s22030750.

[6] A. Kumar, M. Ricketts, S. Hirano, "Ex situ evaluation of nanometer range gold coating on stainless steel substrate for automotive polymer electrolyte membrane fuel cell bipolar plate" *J. Power Sources* **2010**, *195*, 1401–1407. 10.1016/j.jpowsour.2009.09.022.

[7] N. Rojas, M. Sánchez-Molina, G. Sevilla, E. Amores, E. Almandoz, J. Esparza, M. R. Cruz Vivas, C. Colominas, "Coated stainless steels evaluation for bipolar plates in PEM water electrolysis conditions" *Int. J. Hydrogen Energy* **2021**, *46*, 25929–25943. 10.1016/j.ijhydene.2021.03.100.

[8] A. Kellenberger, N. Vaszilcsin, D. Duca, M. L. Dan, N. Duteanu, S. Stiber, T. Morawietz, I. Biswas, S. A. Ansar, P. Gazdzicki, F. J. Wirkert, J. Roth, U. Rost, M. Brodmann, A. S. Gago, K. A. Friedrich, "Towards Replacing Titanium with Copper in the Bipolar Plates for Proton Exchange Membrane Water Electrolysis" *Materials* **2022**, *15*, 1628. 10.3390/ma15051628.

[9] W. Sun, Y. Lv, J. Gao, Q. Feng, B. Jia, F. Ma, "Highly conductive and corrosion-resistant NbN coatings on Ti bipolar plate for proton exchange membrane water electrolysis" *J. Mater. Sci. Technol.* **2025**, *210*, 86–96. 10.1016/j.jmst.2024.05.038.

[10] A. S. Gago, S. A. Ansar, B. Saruhan, U. Schulz, P. Lettenmeier, N. A. Cañas, P. Gazdzicki, T. Morawietz, R. Hiesgen, J. Arnold, K. A. Friedrich, "Protective coatings on stainless steel bipolar plates for proton exchange membrane (PEM) electrolysers" *J. Power Sources* **2016**, *307*, 815–825. 10.1016/j.jpowsour.2015.12.071.

[11] S. Lædre, L. Mendizabal, O. E. Kongstein, A. Oedegaard, H. Karoliussen, F. Seland, "Ta-ITO Coated Titanium Bipolar Plates for Proton Exchange Membrane Water Electrolyzers" *J. Electrochem. Soc.* **2022**, *169*, 034504. 10.1149/1945-7111/ac56a3.
[12] K. Shi, X. Li, Y. Zhao, W.-W. Li, S.-B. Wang, X.-F. Xie, L. Yao, J. O. Jensen, Q.-F. Li, "Corrosion Behavior and Conductivity of TiNb and TiNbN Coated Steel for Metallic Bipolar Plates" *Appl. Sci.* **2019**, *9*, 2568. 10.3390/app9122568.
[13] Z. Zhao, D. Yang, W. Gao, H. Wang, Y. Fu, N. Huang, "Preparation and Performance of Conductive Ti4O7 Coatings on SS316L Bipolar Plates" *J. Therm. Spray Technol.* **2024**, *34*, 301–315. 10.1007/s11666-024-01888-z.
[14] H. Wang, M. A. Sweikart, J. A. Turner, "Stainless steel as bipolar plate material for polymer electrolyte membrane fuel cells" *J. Power Sources* **2003**, *115*, 243–251. 10.1016/s0378-7753(03)00023-5.
[15] L. Fiedler, T. C. Ma, B. Fritsch, J. H. Risse, M. Lechner, D. Dworschak, M. Merklein, K. J. J. Mayrhofer, A. Hutzler, "Stability of Bipolar Plate Materials for Proton-Exchange Membrane Water Electrolyzers: Dissolution of Titanium and Stainless Steel in DI Water and Highly Diluted Acid" *ChemElectroChem* **2023**, *10*, e202300373. 10.1002/celc.202300373.
[16] A. Kraytsberg, M. Auinat, Y. Ein-Eli, "Reduced contact resistance of PEM fuel cell's bipolar plates via surface texturing" *J. Power Sources* **2007**, *164*, 697–703. 10.1016/j.jpowsour.2006.11.033.
[17] K. M. Kim, S. N. Kim, J. H. Kim, Y. Y. Lee, K. Y. Kim, "Study on surface topography of 446M stainless steel as a bipolar plate on interfacial contact resistance of polymer electrolyte membrane fuel cell" *J. Power Sources* **2012**, *220*, 42–46. 10.1016/j.jpowsour.2012.07.102.
[18] U.S. Department of Energy (DOE), "*DOE Technical Targets for Polymer Electrolyte Membrane Fuel Cell Components*", can be found under https://www.energy.gov/eere/fuelcells/doe-technical-targets-polymer-electrolyte-membrane-fuel-cell-components, (accessed: 07/05/2025).
[19] D. Göhl, H. Rueß, A. M. Mingers, K. J. J. Mayrhofer, J. M. Schneider, M. Ledendecker, "Electrochemical Passivation Properties of Valve Transition Metal Carbides" *J. Electrochem. Soc.* **2022**, *169*, 011502. 10.1149/1945-7111/ac47e6.
[20] M. Pourbaix, *Atlas of electrochemical equilibria in aqueous solutions*, National Association of Corrosion Engineers, Houston, **1974**.
[21] U.S. Department of Energy (DOE), "*Technical Targets for Proton Exchange Membrane Electrolysis*", can be found under https://www.energy.gov/eere/fuelcells/technical-targets-proton-exchange-membrane-electrolysis, (accessed: 20/10/2025).
